# A Review of Simultaneous Localization and Mapping for the Robotic-Based Nondestructive Evaluation of Infrastructures

**DOI:** 10.3390/s25030712

**Published:** 2025-01-24

**Authors:** Ali Ghadimzadeh Alamdari, Farzad Azizi Zade, Arvin Ebrahimkhanlou

**Affiliations:** 1 Department of Mechanical Engineering and Mechanics (MEM), Drexel University, 3141 Chestnut St., Philadelphia, PA 19104, USA; 2 Mechanical Engineering Department, Ferdowsi University of Mashhad, Mashhad 9177948944, Iran; 3 Department of Civil, Architectural and Environmental Engineering (CAEE), Drexel University, 3141 Chestnut St., Philadelphia, PA 19104, USA

**Keywords:** SLAM, GPS-denied, vision-based, LiDAR-based, LiDAR-visual-inertial, localization, benchmarking, robotic inspection, infrastructure

## Abstract

The maturity of simultaneous localization and mapping (SLAM) methods has now reached a significant level that motivates in-depth and problem-specific reviews. The focus of this study is to investigate the evolution of vision-based, LiDAR-based, and a combination of these methods and evaluate their performance in enclosed and GPS-denied (EGD) conditions for infrastructure inspection. This paper categorizes and analyzes the SLAM methods in detail, considering the sensor fusion type and chronological order. The paper analyzes the performance of eleven open-source SLAM solutions, containing two visual (VINS-Mono, ORB-SLAM 2), eight LiDAR-based (LIO-SAM, Fast-LIO 2, SC-Fast-LIO 2, LeGO-LOAM, SC-LeGO-LOAM A-LOAM, LINS, F-LOAM) and one combination of the LiDAR and vision-based method (LVI-SAM). The benchmarking section analyzes accuracy and computational resource consumption using our collected dataset and a test dataset. According to the results, LiDAR-based methods performed well under EGD conditions. Contrary to common presumptions, some vision-based methods demonstrate acceptable performance in EGD environments. Additionally, combining vision-based techniques with LiDAR-based methods demonstrates superior performance compared to either vision-based or LiDAR-based methods individually.

## 1. Introduction

This paper aims to study and evaluate the performance of simultaneous localization and mapping (SLAM) methods in enclosed and GPS-denied (EGD) environments, with a particular emphasis on robotic infrastructure inspection [1,2,3,4,5]. The term “enclosed” specifically refers to environments characterized by confined or restricted spatial characteristics, such as inside bridges [6], tunnels [7], pipeline housings [8], and underground utilities [9]. These environments may be poorly lit, meaning they lack adequate external light sources and rely solely on the moving platform’s onboard lighting systems for illumination. EGD environments may also be feature-poor, encompassing areas with heterogeneous textures, repeating patterns, or a scarcity of distinct visual cues that challenge sensor perception. “GPS-denied” highlights environments where global positioning systems are ineffective due to obstructions or signal blockages.

SLAM in EGD settings plays a critical role in infrastructure inspection. It is also crucial for construction site monitoring, asset management, and urban planning, where precise mapping and localization are essential. Furthermore, SLAM technologies are invaluable in operations like search and rescue missions following hazardous incidents or explorations in environments inhospitable to humans. While SLAM can be applied across various domains, including airborne, underwater, and terrestrial environments, this study specifically focuses on ground-based SLAM within EGD settings, highlighting its significance in enhancing the safety, efficiency, and effectiveness of infrastructure-related activities.

SLAM techniques process data from sensors to simultaneously create an environmental map and localize the platform within it. These techniques are applicable to diverse moving platforms, such as vehicles, robots, or hand-held devices equipped with the necessary sensors. For simplicity, all such platforms will be referred to as moving platforms in this paper. Primary 3D SLAM methods have evolved alongside technological advancements, resulting in significant paradigm shifts and breakthroughs in the field [10,11,12,13]. SLAM techniques vary based on the types of sensors used, each possessing distinct strengths and limitations that must be carefully considered for optimal application selection.

Light detection and ranging (LiDAR)-based SLAM uses laser measurements to capture 3D point clouds that facilitate real-time topology and surface reconstruction [14]. Due to its robustness in diverse lighting and feature-poor conditions, LiDAR is widely utilized in EGD settings. It is generally accepted that LiDAR-based methods deliver superior localization accuracy compared to vision-based approaches [15,16]. On the other hand, vision-based SLAM uses camera visionary data to capture the environment and detect its available features. In comparison, LiDAR-based SLAM produces a high-accuracy map of the surrounding environment. However, it is more expensive and consumes more power compared to vision-based methods. Moreover, LiDAR-based SLAM is computationally demanding when used to produce occupancy maps for 3D large-scale environments [16]. On top of that, transparent and translucent objects are invisible to LiDAR sensors [17]. Compared to LiDAR-based methods, vision-based SLAM consumes less power, is cost-effective, and its equipment is lightweight. However, it tends to fail in severe motion or feature-poor environments with poor lighting conditions and produces less dense point clouds [18]. Depending on the application, LiDAR sensors and cameras may be used alone or in combination with other measurement sources, such as inertial measurement units (IMU) and encoders.

Vision-based SLAM is further divided into visual SLAM and visual-inertial SLAM, while LiDAR-based SLAM includes LiDAR SLAM and LiDAR-inertial SLAM. Both vision-inertial and LiDAR-inertial techniques can be classified as tightly coupled or loosely coupled based on their data fusion approach. Hybrid SLAM methods combine LiDAR and camera data, enhancing odometry accuracy through the integration of LiDAR’s precise depth measurements with the rich visual texture data from cameras.

Visual-inertial and LiDAR-inertial methods, respectively, combine camera and LiDAR data with IMU data to obtain more accurate odometry results compared to their corresponding singular data source methods. Due to a relatively low price, camera(s) are already available on most moving platforms. Additionally, the cost of adding a camera to a LiDAR-based SLAM is negligible compared to the required equipment in a LiDAR-based SLAM. However, it can bring about a superior optimized trajectory [18]. For example, a combination of LiDAR-based and vision-based SLAM [19] could result in even higher accuracy in odometry using the rich visual texture information and accurate LiDAR depth measurements on outdoor environment datasets with substantial features such as the KITTI dataset [20].

SLAM has been extensively studied over recent years, with numerous surveys exploring a broad spectrum of SLAM methodologies. To provide context for this review, Table 1 summarizes the key review papers published from 2008 onwards, categorizing them based on their focus areas, including vision-based methods, LiDAR-based methods, and GPS-denied environments. Earlier reviews from before 2008 are also included and covered in these surveys. This overview in Table 1 highlights the evolution and breadth of SLAM research, forming the foundation for the present analysis.

One of the early surveys published in 2008 reviewed filtering methods in SLAM, aiming to reduce the computational burden by minimizing the data required for processing large-scale environments [21]. However, this review lacks coverage of challenging environments where landmark detection is difficult. In 2011, a review focused on visual odometry, a key component in pose estimation within SLAM, discussing methods utilizing monocular and stereo cameras [22]. This was followed by a 2012 review from the same authors that expanded on feature detection and tracking in sequential frames, including optimization methods for position estimation [23].

In 2015, two significant reviews examined visual SLAM. The first covered RGB-D methods and the core components of SLAM, such as localization and mapping [24], while the second focused on feature detection and image matching for loop closure detection, omitting the mapping aspect [25]. To provide a comprehensive review of SLAM mapping, a survey in the same year addressed methods for representing the surrounding environment, utilizing visual and other sensor measurements [26]. This survey covered various scene descriptors, including global descriptors, local feature-based descriptors, and hybrid approaches.

A broad review published in 2016 addressed key SLAM elements, such as robustness in harsh environments, scalability, and representation methods for 2D and 3D environments [27]. In 2017, reviews explored visual SLAM techniques like key-frame-based methods with monocular cameras, identifying areas for improvement in vision-based SLAM [28]. Another study in the same year categorized visual SLAM into feature-based, direct methods, and RGB-D SLAM while also outlining open challenges [29]. The focus on vision-based SLAM in dynamic environments emerged in 2018, with reviews discussing structure from motion (SfM) and categorizing visual SLAM methods into direct and indirect approaches [30,31]. Additionally, a 2018 survey examined visual-inertial SLAM, detailing filtering and optimization techniques and providing insights into SLAM trends of that period [32].

Beginning in 2019, the integration of machine learning (ML) and artificial intelligence (AI) in SLAM became increasingly prominent. A 2019 survey highlighted the application of ML in scene recognition and localization [33]. In 2020, a review explored the use of deep reinforcement learning (DRL) in vision-based SLAM, categorizing approaches into various navigation frameworks [34]. Sensor fusion also gained attention, with a 2020 survey covering visual, LiDAR-based, and visual-LiDAR fusion SLAM techniques [16].

Subsequent reviews in 2020 and 2021 delved into specific aspects such as feature-based visual SLAM, radio frequency-based localization, and sensor fusion [18,35,36]. A 2021 study provided a comparative analysis of popular SLAM algorithms on benchmark datasets [36]. The role of loop closure detection in SLAM was also explored, particularly focusing on deep learning-based approaches [37].

The trend of sensor fusion methods continued into 2022, with reviews examining multi-sensor data fusion in SLAM, its theories, and algorithmic categorization [41]. Another review addressed SLAM applications in subterranean environments, highlighting system architectures and platform specifics [39]. In 2022, a study reviewed LiDAR-based multi-sensor fusion SLAM, focusing on tightly and loosely coupled methods and comparing performance using standardized datasets [40]. The latest reviews in 2022 provided insights into learning-based methods for scene understanding in SLAM, reflecting the evolution of SLAM technologies [38,42].

**Figure 1:**
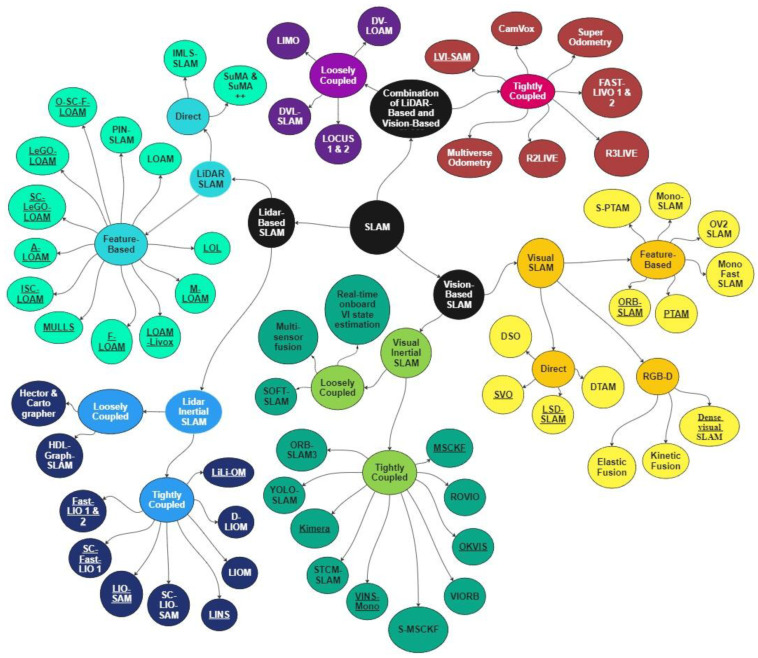
Classification of SLAM methods. Previous surveys. Methods with an underline are considered for benchmarking.

In 2023 and 2024, researchers primarily concentrated on SLAM challenges caused by environments. Recent surveys have explored various aspects, including the semantic understanding of environments [43], dynamic environments [44], and extreme environments [39]. Despite these advancements, a significant gap persists in reviewing SLAM methods specifically tailored for EGD environments, particularly with benchmarking. While several surveys on SLAM techniques exist, a systematic evaluation of performance improvements through combined vision-based and LiDAR-based methods in EGD environments remains underexplored. This paper aims to address this gap by comprehensively reviewing and benchmarking SLAM methods designed for EGD environments, with a focus on infrastructure inspection and maintenance applications. In this paper, we focus on well-known SLAM algorithms that exhibit a clear evolution over time and have made a remarkable impact on the field. Our selection and categorization were conducted chronologically by tracing each method from previous surveys and original sources. We considered SLAM algorithms that are open-source and accompanied by peer-reviewed publications or technical reports. Only algorithms with consistent ROS-based packages are included in the benchmarking process. To facilitate benchmarking, we utilize our dataset and a publicly available dataset from EGD conditions. Figure 1 illustrates the classification of reviewed methods and their benchmarking status. Methods with an underline are considered for benchmarking.

The paper is structured as follows. Section 2 presents the problem statement and identifies common elements in SLAM methods. Section 3 and Section 4 delve into vision-based SLAM and LiDAR-based SLAM, respectively, discussing their methodologies, advantages, and limitations. Section 5 explores the integration of LiDAR and vision-based techniques, highlighting their combined benefits. Section 6 focuses on NeRF-based SLAM. Section 7 presents the benchmarking process, providing comparative analysis and results. Finally, Section 8 concludes the paper, summarizing the findings and discussing future research directions.

## 2. Problem Statement and Common Elements

SLAM refers to the ability of a moving platform to construct a map of its surroundings while concurrently estimating its location within the generated map [45]. SLAM solutions are critical in various applications, including autonomous navigation, robotics, and augmented reality, where the platform must operate in diverse and often challenging environments. These environments may include enclosed spaces, outdoor areas, feature-poor settings, poorly lighted conditions, and GPS-denied zones. Operating in enclosed or GPS-denied environments, such as underground mines, tunnels, or indoor spaces, presents significant challenges due to the lack of reliable positioning data from satellite systems. Outdoor environments can introduce additional complexities, such as varying light conditions and unstructured terrain, which may adversely affect sensor performance and feature detection. In feature-poor settings, such as empty corridors or open fields, traditional SLAM algorithms struggle due to insufficient distinguishing landmarks, leading to localization errors and map inaccuracies. Poorly lighted environments further exacerbate these issues by reducing the effectiveness of vision-based methods, necessitating the integration of additional sensors or advanced image processing techniques to maintain reliable performance. This section explores the common technical concepts associated with SLAM solutions, categorizing elements into vision-based and LiDAR-based methods, along with shared components between these approaches. The discussion also addresses potential malfunctions and limitations of SLAM in challenging conditions, providing a comprehensive overview of the state-of-the-art methods and their operational constraints.

### 2.1. Vision-Based

The EGD settings may present near-total darkness, warranting the exclusion of vision-based techniques [16,38,39]. However, artificial lighting or robotic platform lighting might be used to solve the problem. Therefore, we also considered vision-based methods in this study.

#### 2.1.1. Visual Feature Detection and Tracking

The purpose of detecting and tracking features in vision-based SLAM is to compute the relative position and orientation of a moving platform in space. A salient feature is a distinct point or pattern in an image that stands out from its surroundings. Corners and edges are commonly used as salient features of landmarks in vision-based systems due to their invariant properties to lighting, orientation, and scale changes. A feature can include points, regions, or segments extracted from a frame. The most common feature types used in vision-based methods are Harris [46], features from accelerated segment test (FAST) [47], scale-invariant feature transform (SIFT) [48], speeded-up robust features (SURF) [49], and oriented fast and rotated brief (ORB) [50]. SIFT performs best in most scenarios, while ORB is the fastest algorithm. SURF and ORB outperform SIFT in the case of rotation angles of 90 degrees, and ORB shows almost similar performance to SIFT in noisy images [51].

Reference [18] classified feature detectors into three main categories: low-level, high-level detectors, and a combination of these two methods. Low-level feature detectors include detecting points, edges, and planes, while high-level detectors use semantically labeled objects. High-level detectors use both descriptors (are described in common between vision-based and LiDAR-based) and data association methods to define objects in a scene. References [52,53,54,55,56,57] are examples of SLAM approaches that utilize low-level feature detectors for localization and mapping. References [58,59,60,61,62,63,64,65,66,67] utilize high-level feature detectors for navigating in both static and dynamic environments. Within the third category, references [67,68,69,70,71,72,73,74,75,76] benefit from the combination of low-level and high-level feature detectors.

It is possible to compute localization by tracking the movement of the detected features in consecutive frames. To track the position of the feature over consecutive frames, four types of descriptor matching [77], filter-based tracking [78], optical flow tracking [79] and direct pixel processing [80] methods exist.

#### 2.1.2. Structure from Motion and Visual Odometry

**Figure 2:**
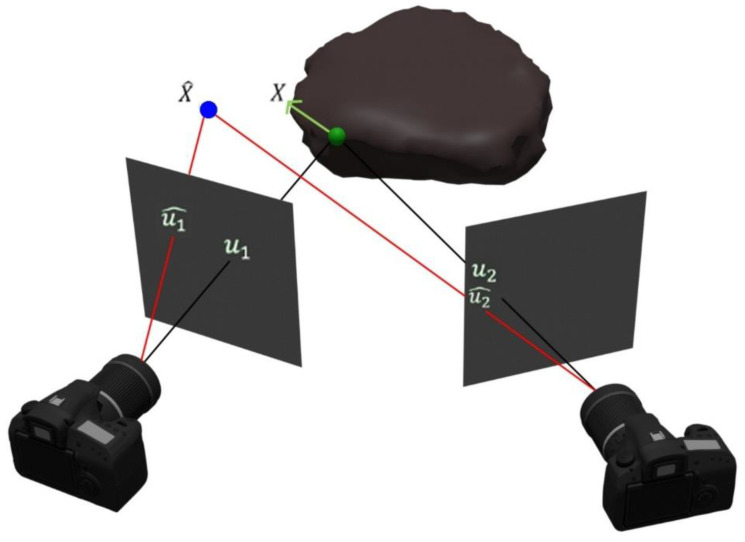
Triangulation and minimizing reprojection error in SfM.

SfM is a technique that uses 2D images from multiple views to build a 3D structure of a scene [81]. Equation (1) is the relationship between a 3D point $\hat{X}$ in space and the pixel position u in a 2D image array:

(1) $$\tilde{u}=P\tilde{X}$$

where P is the projection matrix. As depicted in Figure 2, SfM projects the detected feature in a sequence of views into the camera plane. However, the back-projected rays do not intersect on the actual point position in 3D space because of measurement noise. Thus, intersections between back-projected rays are rare. Squared error is used as an error metric for comparing the predicted and the actual projected point on the camera plane. Based on that, the feature point that has the lowest squared error is considered in the next view.

(2) $$X=argmin_{X}\sum_{i}{\left(u_{i}-\tilde{u_{i}}\left(P_{i},X\right)\right)}^{2}$$

where $u_{i}$ and $\tilde{u_{i}}\left(P_{i},X\right)$ are actual and predicted point position in view i. SfM uses the triangulation [82] method to obtain the position of the camera in a sequence of images. Reference [83] introduced an SfM method that can run in real-time, and reference [84] introduced incremental SfM, named visual odometry. Similarly, visual odometry is a technique to estimate the position of a moving platform periodically. According to the data collected from the camera, the method acquires the relative movement of a moving platform [24,84]. In other words, visual odometry is a sub-category of real-time SfM.

#### 2.1.3. Direct Photometric Error

In visual odometry, direct photometric error is used to estimate the position and orientation of a camera by minimizing the difference in pixel brightness between two images.

### 2.2. LiDAR-Based

#### 2.2.1. Scan Matching

A scan-matching algorithm finds the transformation between two positions or orientations of a moving platform based on consecutive scans. The iterative closest point (ICP) [85] algorithm is the widely used method for scan matching. ICP aligns a sequence of scanned point clouds by finding the target’s closest points and minimizing the corresponding points’ distances [86]. There are several types of ICP algorithms including point-to-point [87], point-to-surface [88], generalized ICP (GICP) [89], voxelized GICP (V-GICP) [90], point-to-plane iterative closest point (P2P-ICP) [91], color iterative closest point (ColorICP) [92], and 4 dimensional (4D) ICP [93]. When the number of points grows, ICP becomes computationally inefficient. Thus, reference [94] proposed an algorithm to enhance the efficiency in high-resolution data. Among the most recent ICP methods are keep-it-small-and-simple ICP (KISS-ICP) [95], continues-time ICP (CT-ICP) [96], extreme (X)-ICP [97], generalized (G)-ICP [89], and GenZ-ICP [98]. The KISS-ICP proposed an ICP with an adaptive threshold to remove outliers based on initial pose error, sensor noise, number of dynamic objects, and type of dynamic objects. It also uses a constant velocity model [99] for motion prediction and de-skewing scans. As its name suggests, the constant velocity model assumes the last step velocity for the following steps. The CT-ICP proposed a LiDAR odometry that took both pose continuity within scans and scan discontinuities into account. Moreover, the X-ICP introduced a localizability-aware module that enhances the robustness and accuracy of LiDAR-based localization in challenging environments [97]. This module analyzes the localizability of the environment and applies constraints based on this analysis, improving pose estimation, and ensuring reliable localization even in extreme and geometrically uninformative conditions. Additionally, G-ICP enhances traditional ICP by integrating point-to-point and point-to-plane metrics into a probabilistic framework, leveraging point covariances for better surface modeling [89]. This can result in more accurate and robust alignment, especially in complex or noisy environments. Lastly, GenZ-ICP is an advanced ICP method for LiDAR-based odometry that combines point-to-plane and point-to-point error metrics with adaptive weighting based on environmental geometry [98]. GenZ-ICP aims to enhance adaptability and resilience, particularly in corridor-like scenarios.

Normal distribution transformation (NDT) [100] is another common point-matching method. First, the method creates probability function grids of the point cloud data and calculates their local distributions. Then, it matches points to the normal distributions, which improves computational efficiency. However, the accuracy of the NDT depends on the grid size (a larger grid means worse accuracy) [101].

In comparison, ICP is more sensitive to initial alignment, while NDT’s probability nature makes it more robust to noise [102]. Lastly, ICP is more common in LiDAR-based methods but can be used in vision-based methods too, such as Kinect Fusion methods [103].

#### 2.2.2. LiDAR Feature Points

In most LiDAR-based methods, feature points are extracted from sharp edges and planar surfaces in the point clouds based on local surface smoothness. However, some methods may define diverse types of features such as ceiling, pillar, beam, and vertex [104]. The goal is to obtain trajectory by tracking features.

**Figure 3:**
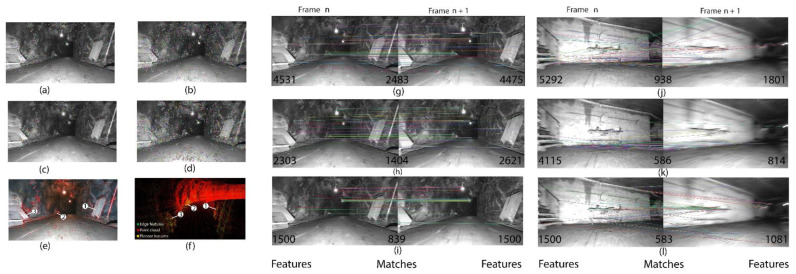
Visual feature types, LiDAR point cloud, feature types, and tracking loss. (**a**) SURF. (**b**) SIFT. (**c**) ORB. (**d**) Harris. (**e**) FAST. (**f**) Point cloud and detected features. (**g**) SURF. (**h**) SIFT. (**i**) ORB feature matching. (**j**) Tracking loss in severe rotation for SURF feature matching. (**k**) Tracking loss in severe rotation for SIFT feature matching. (**l**) Tracking loss in severe rotation for ORB feature matching (colors of the points and lines are intended to differentiate independent points and lines in (**a**–**e**,**g**–**l**). In (**f**), green and yellow points, respectively, represent edge and planar features, while other points are red).

Figure 3 represents five main types of vision features illustrated in Figure 3a–e, LiDAR point cloud along with its edge and planar features is depicted in Figure 3f, and success and failure in feature tracking is presented in Figure 3g–l. Each pair of pictures in feature tracking includes a number of detected features in frame number n and consecutive frame n+1. As depicted in Figure 3g–i, the number of SURF features in Figure 3g in a frame is higher compared to other features, resulting in a more intensive matching process. In Figure 3j–l, the ORB-based method in Figure 3l detects fewer features but matches a higher ratio compared to SIFT in Figure 3k and SURF in Figure 3j. In general, poor lighting conditions intensify blur image occurrence in severe rotations, which cause fewer feature detections and wrong matches.

### 2.3. Optimization and Mapping in SLAM Systems

The simultaneous localization and mapping (SLAM) process integrates various algorithms and methodologies to enable autonomous systems to build maps of unknown environments while simultaneously determining their position within them. As the complexity and scale of the environment increases, the SLAM system must address challenges such as accumulated errors, data fusion, and efficient representation of spatial information. This section provides an overview of key concepts and techniques used in SLAM, organized into thematic categories that reflect their primary functions and roles in the SLAM pipeline.

#### 2.3.1. Optimization Techniques

Optimization is crucial in SLAM, particularly when large-scale environments introduce significant accumulated errors in the constructed map. Graph optimization techniques minimize these errors by adjusting the previously estimated positions of the moving platform, especially when a loop closure event is detected. Optimization can be divided into front-end and back-end processes, where the front end focuses on feature detection and map construction, and the back-end handles pose optimization for precise localization. Notable optimization libraries include Georgia Tech smoothing and mapping (GTSAM), general graph optimization (g2o), and Ceres solver, which support the implementation of various optimization algorithms, including incremental smoothing and mapping [105,106].

#### 2.3.2. State Estimation and Filtering Methods

Accurate state estimation is fundamental to maintaining reliable localization and minimizing drift over time. Kalman filter (KF) methods, including extended Kalman filter (EKF) and unscented Kalman filter (UKF), play a critical role in estimating unknown variables from noisy sensor measurements. These filters combine state and observation models to predict system states, effectively accounting for sensor inaccuracies. EKF and UKF are specifically designed to handle nonlinearities in state and measurement models, making them suitable for complex real-world applications [107,108].

#### 2.3.3. Sensor Fusion Techniques

SLAM systems often rely on sensor fusion techniques to combine data from multiple sensors, improving localization accuracy and map consistency. Sensor fusion can be implemented through tightly or loosely coupled methods. Loosely coupled systems independently process each sensor’s data and fuse the results, providing simplicity and ease of development but at the cost of accuracy and computational efficiency. In contrast, tightly coupled systems jointly optimize sensor data, making them robust to signal variances and external perturbations, though they require more complex and computationally demanding implementations [79,109].

#### 2.3.4. Map Representations and Spatial Data Structures

Various data structures and representations are employed to model the environment within SLAM systems. Point clouds, voxel grids, and surfels offer different methods to capture and represent spatial information, with each approach suited to specific applications. K-dimensional (K-D) trees enable efficient searching and manipulation of multi-dimensional data, while descriptors such as local and global descriptors provide high-level abstractions of geometric features. Additionally, pose graphs model spatial constraints and relationships between sequential poses, which are critical for loop closure detection and global map consistency [110,111].

#### 2.3.5. Loop Closure and Outlier Rejection

Loop closure detection is essential for recognizing previously visited locations and correcting accumulated drift errors. Vision-based SLAM methods commonly utilize feature matching and bag of words (BoW) techniques for identifying revisited scenes, while LiDAR-based methods employ various descriptors. The random sample consensus (RANSAC) algorithm aids in the robust estimation of geometric models by rejecting outliers in data, enhancing the accuracy of loop closure corrections and environmental map construction [112,113].

#### 2.3.6. Specialized Techniques and Adjustments

SLAM systems often incorporate specialized techniques such as bundle adjustment, sliding window methods, and extrinsic parameter estimation to refine and optimize performance. Bundle adjustment integrates data from multiple sensors and aligns them to improve spatial consistency, while sliding window techniques optimize computational efficiency by focusing only on recent measurements. Extrinsic calibration aligns the relative positioning and orientation of different sensors, ensuring that fused data accurately represent the environment [114,115].

### 2.4. SLAM Evaluation Metrics

#### 2.4.1. Tracking Loss

The tracking loss problem occurs when the platform moves too fast or there are some disturbances, such as dust, in the environment. The process of calculating camera position with respect to the map is called re-localization. The problem of tracking loss and the corresponding position loss on the map is also called the kidnapped robot problem in robotics.

#### 2.4.2. Drift Error

**Figure 4:**
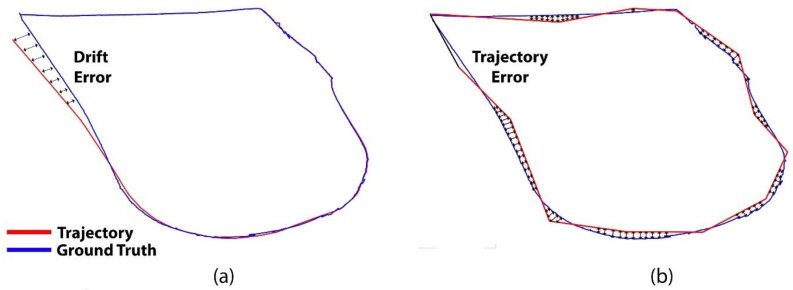
Drift and trajectory error during path traversal. (**a**) Drift error at the end of the traversed path. (**b**) Trajectory error during the path traversal.

The uncertainty and accumulated error grow by traversing large distances considering that no loop closure detection happens. In case of no absolute positioning sensor such as GPS, the localization depends on a relative positioning system that relies on IMU, LiDAR, camera, etc. Thus, drift error occurs in such systems in case of wheel slippage, noisy IMU data, and feature tracking errors [116]. To reduce drift error, most systems rely on loop closure detection. Figure 4a depicts drift error.

#### 2.4.3. Translation Error

Translation error is defined as the difference between the estimated position and ground truth position in each frame.

#### 2.4.4. Trajectory and Traversing Error

The absolute trajectory error is the average deviation from the ground true trajectory per frame [117]. The root mean square error (RMSE) is the most popular index of absolute trajectory error. It evaluates the performance of various methods according to the available ground truth. Traversing error, on the other hand, refers to the error in the distance traveled by a robot along a straight-line path. This can occur due to factors such as wheel slippage, encoder inaccuracies, or uneven terrain. Traversing error is typically measured by comparing the actual distance traveled by the robot along a straight line to the intended or planned distance and calculating the difference between them. The overall trajectory error is represented in Figure 4b.

#### 2.4.5. Point Cloud Distortion Error

LiDAR data are often collected in the form of point clouds, which are datasets containing points in three-dimensional space. Point cloud distortion means that the points in the cloud do not follow the measurement pattern of the sensor. This can have a significant impact on the accuracy of subsequent analyses using this data. LiDAR point clouds are prone to distortion due to moving objects and the movements of the platform during measurements. SLAM algorithms often compensate for distortion using IMU data [118].

## 3. Vision-Based SLAM

Vision-based SLAM methods utilize various cameras, such as stereo [119], red–green–blue–depth (RGB-D) [120], and monocular cameras. They can also take other sensor inputs, including IMU, wheel encoder, and optional global positioning for localization [25]. This study classifies vision-based SLAM into two categories: visual SLAM and visual-inertial SLAM. Visual SLAM methods use only camera input for localization and mapping, while visual-inertial SLAM methods combine vision sensors with IMU inputs to achieve precise localization. Visual-inertial SLAM methods fall into two sub-categories, including tightly and loosely coupled methods according to their sensor fusion type. Most visual SLAM tends to fail in severe rotations because of blur images occurring or under/over exposure [121], while visual-inertial SLAM methods are more resistant to severe rotations due to IMU compensation.

### 3.1. Visual SLAM

Visual SLAM combines visual odometry with loop closure detection by using map optimization methods to refine the global map. Specifically, these methods use loop closure information to correct errors in the map and improve the accuracy of the visual odometry estimates [22,23]. Generally, visual SLAM utilizes visual odometry methods for localization combined with global map optimization to build a consistent geometric map of the environment [122,123]. This study classifies the visual SLAM methods into three main sub-categories: feature-based, direct, and RGB-D.

#### 3.1.1. Feature-Based Methods

**Figure 5:**
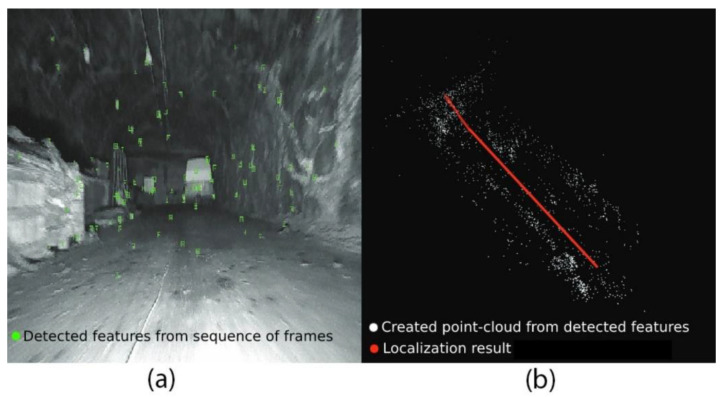
Visual SLAM feature-based method workflow demonstration. (**a**) Feature tracking using the camera. (**b**) 3D mapping using the detected features.

**Figure 6:**
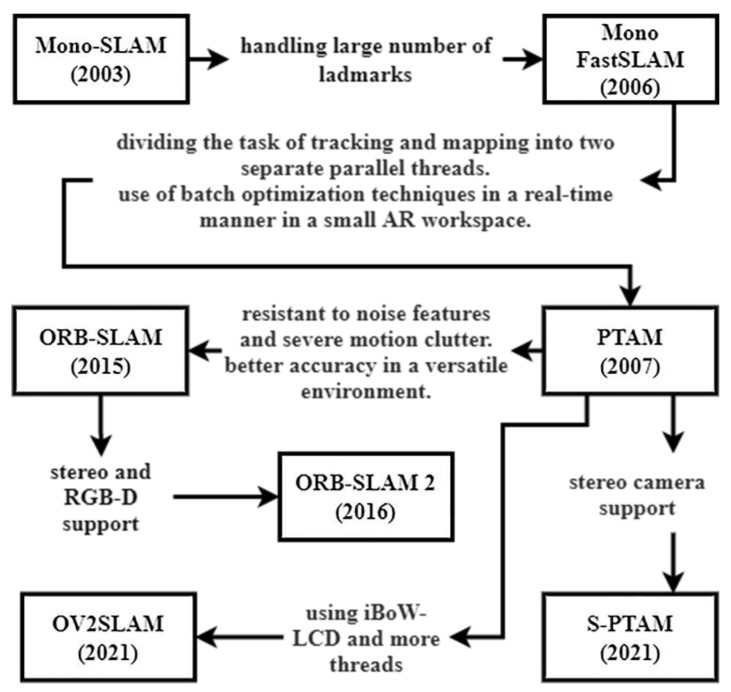
Evolution of visual feature-based methods.

Feature-based methods detect and track available features in the environment to obtain localization and build the 3D structure of the environment. Feature-based methods are usually less computationally expensive compared to direct and RGB-D methods due to using feature points instead of tracking a large number of pixels on the frame. However, the point cloud density created by feature-based methods relies on the available salient features of the environment. Figure 5 depicts the process of detecting distinct points or patterns in 2D frames and matching them across multiple frames to create a 3D point cloud using features such as SIFT or SURF. Triangulation techniques are applied to estimate the depth of each point in 3D space, and the resulting point cloud can be used for 3D mapping. The following covers some of the visual feature-based SLAM methods. Figure 6 shows the evolution of feature-based methods.

- Mono-SLAM

Mono-SLAM was the first sole vision-based approach, which was introduced in 2003 [124,125]. Mono-SLAM utilizes features with high information content and distinctive visual characteristics within a frame for navigation and mapping in the environment. These features are typically robust and repeatable across multiple frames, enabling accurate and reliable localization and mapping. Equation (2) uses high-quality features that are close together in consecutive frames by a threshold. Mono-SLAM generates a probabilistic feature-based map within which each feature estimation contains a probability of its presence. However, the method cannot explore large-scale environments because computational complexity increases with a time quadratic equation. The maximum number of features Mono-SLAM can process is also limited to 100 using 30 frames per second (fps) video streams [124].

The state vector at time k is denoted as $x_{k}$ and is defined as:

(3) $$x_{k}=\left[r_{k},f_{1},f_{2},\ldots ,f_{n}\right]$$

where $r_{k}$ is the 6D pose of the camera at time k, represented as a vector $\left[x_{k},y_{k},z_{k},q_{k}\right]$, and $f_{i}$ is the 3D position of the ith feature in the scene.

The system dynamics describe how the state vector evolves over time in response to control inputs $u_{k}$ and process noise $w_{k}$:

(4) $$x_{k}+1=g\left(x_{k},u_{k}\right)+w_{k}$$

The observation model predicts the 2D image coordinates of the features given the current camera pose and feature positions:

(5) $$z_{k}=h\left(x_{k}\right)+v_{k}$$

The goal of Mono-SLAM is to estimate the maximum a posteriori (MAP) state vector $x_{h}at_{k}$ given the measurements up to time k:

(6) $$\hat{x_{k}}=argmaxP\left(x_{k}|z_{1},z_{2},\ldots ,z_{k}\right)$$

Mono-SLAM uses the Kalman filter to solve the above estimation problem.

- Mono Fast SLAM

The earlier EKF-based SLAM approaches could handle a limited number of landmarks in the environment due to computational complexities. To address the issue, Mono Fast SLAM was introduced in 2006, which is a probabilistic approach to SLAM, which uses a camera as the only sensor [126]. Additionally, sensor updates required time quadratic in the number of landmarks K to compute. These complexities stem from the fact that the covariance matrix maintained by the Kalman filters has $O\left(K2\right)$ elements, all of which must be updated even if just a single landmark was observed [127]. To solve the problem, Mono Fast SLAM managed to use vision-based SLAM combined with particle filtering methods for large-scale environments.

- PTAM

**Figure 7:**
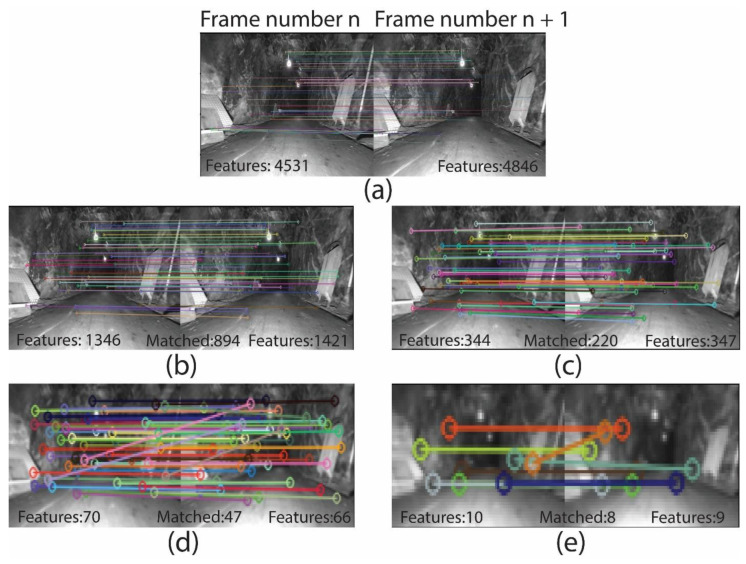
Tracking in a four-level pyramid in PTAM method. The images include a maximum of 50 closest features in consecutive frames. (**a**) original image. (**b**) first level. (**c**) second level. (**d**) third level. (**e**) fourth-level pyramids.

To better optimize the tracking and mapping of the camera pose, parallel tracking and mapping (PTAM) proposed an algorithm that divided the task of tracking and mapping into two separate parallel threads [128]. The method enables real-time batch optimization techniques to be used for small, augmented reality (AR) workspaces, which are used for SLAM in virtual reality (VR) applications. PTAM applies the FAST feature detection algorithm to image pyramids, which are multi-scale representations of an image, to build clusters of corner features. Image pyramids are created by repeatedly downsampling the original image to create smaller versions of it at multiple scales, allowing for the detection of features at diverse levels of detail. Thus, it obtains initial pose estimation using coarse feature matching and optimizes localization using matched features in higher levels of the pyramids, as depicted in Figure 7. PTAM also introduced the key-frame selection method and map initialization using a 5-point algorithm. The PTAM method extends the map incrementally and creates many points on it. Moreover, it uses Equation (1) for projecting points in 3D space and Equation (2) for feature matching and localization. The PTAM method is vulnerable to noise and lacks accuracy when the surrounding scenario changes.

- ORB-SLAM

To address the limitation of PTAM, ORB-SLAM was introduced in 2015. This method produces features that are resistant to noise, robust to severe motion clutter, and maintain accuracy in a versatile environment [55]. ORB-SLAM uses ORB features to track the camera’s movement and build a map of the environment. It operates in real-time and can work with both monocular and stereo cameras. ORB-SLAM combines feature detection, tracking, and mapping using a keyframe-based approach that allows for efficient and robust performance. ORB-SLAM is among the best feature-based monocular visual SLAM systems, which is open-source and ROS-based. Additionally, there is an ORB-SLAM 2 (2016) extension that supports monocular, stereo, and RGB-D cameras [129].

The automatic map initialization of the ORB-SLAM starts with finding initial correspondences. It extracts ORB features of the current scan $\left(F_{c}\right)$ and matches its feature points $\left(x_{c}\right)$ with the feature points $\left(x_{r}\right)$ of the reference frame $\left(F_{r}\right)$. Next, it computes two models in parallel, namely, a homograph $\left(H_{cr}\right)$ and a fundamental matrix $\left(F_{cr}\right)$.

(7) $$x_{c}=H_{cr}x_{r},x_{c}^{T}F_{cr}x_{r}=0$$

To compute $H_{cr}$ and $F_{cr}$, it employs normalized direct linear transform (DLT) and eight-point algorithms [130].

Following that, it selects one of the models according to a robust heuristic, $R_{H}$.

(8) $$R_{H}=\frac{S_{H}}{S_{H}+S_{F}}$$

If $R_{H}>0.45$, it selects homograph. $S_{H}$ and $S_F$ are defined as follows.

(9) $$S_{M}=\sum_{i}\left(p_{M}\left(d_{cr}^{2}\left(x_{c}^{i},x_{r}^{i},M\right)\right)+p_{M}\left(d_{rc}^{2}\left(x_{c}^{i},x_{r}^{i},M\right)\right)\right)$$

(10) $$p_{M}=5.99-d^{2}\;if\;d^{2}<T_{M}\;or\;0\;if\;d^{2}>T_{M}$$

where M indicates the model (H or F), $T_{H}=5.99$, and $T_{F}=3.84$.

After model selection, the ORB-SLAM computes motion and structure from motion recovery. In the case of homograph and fundamental matrix, it relies on Faugeras and Lustman [131] and decomposition [130] methods. Finally, the ORB-SLAM implements bundle adjustment for initial reconstruction refinement.

- S-PTAM

Following the PTAM, stereo parallel tracking and mapping (S-PTAM) proposed the use of stereo constraint in initialization, mapping, and feature tracing processes [132] in 2021. Moreover, it took advantage of an appearance-based matching loop detection within pose graph optimization and used bundle adjustment for map optimization.

- OV2SLAM

In 2021, online and versatile visual SLAM (OV2SLAM) proposed a multi-thread visual SLAM like the PTAM but with four threads [133]. The method used incremental bag-of-words loop closure detection (iBoW-LCD) [134] to enable incremental vocabulary tree building, fitting better to the live environment without offline training biases.

#### 3.1.2. Direct Methods

**Figure 8:**
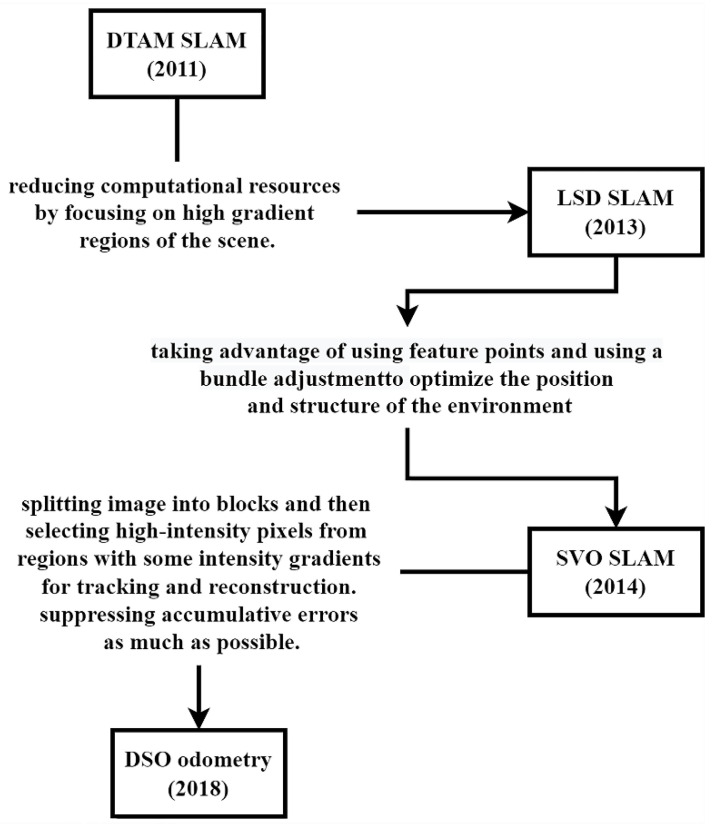
Evolution of direct methods.

The popularity of direct photogrammetric SLAM (or direct SLAM) has increased due to the growth in computational resources and reduced noise in cameras. This approach generates a denser point cloud of the environment, allowing for greater precision in localization, particularly in dynamic settings. Unlike feature-based methods that track the position of detected features in 3D space, direct methods consider pixel properties (such as color, brightness, intensity, and gradient) and monitor the movement of a considerable number of pixels from the region of interest of one frame to another. This section discusses direct methods and provides an evolution summary illustrated in Figure 8.

- DTAM

In 2011, the first direct method, known as dense tracking and mapping (DTAM), was introduced [103]. DTAM is based on keyframes and generates an initial depth map using stereo measurements, taking multiple stereo baselines for each pixel. After selecting a keyframe, the photometric error is calculated for each pixel in consecutive frames to estimate the cost volume. DTAM then tracks camera motion between frames by comparing the image to the model view generated from the map. As each new image frame is processed, depth information for every pixel is estimated and optimized.

- LSD-SLAM

The DTAM technique faced limitations due to its requirement for substantial computational resources. To address the high computational need of DTAM, large-scale direct SLAM (LSD-SLAM) was introduced in 2013. This method concentrated on the high-gradient areas of each scene, particularly edges, and operated in real-time on a central processing unit (CPU) [122]. The minimal resource utilization makes LSD-SLAM a practical approach for real-world large-scale and AR applications.

- SVO

In 2014, semi-direct visual odometry (SVO) was introduced, which focused on precise localization, robustness, and rapid processing [135]. The method uses feature points interleaved with indirect methods but uses the direct method to perform frame-to-frame motion estimation on the tracked features. Direct methods of visual odometry rely on the pixel intensity values in consecutive image frames to estimate motion. This approach eliminates the need for feature extraction and matching, which can be computationally expensive. On the other hand, indirect methods employ feature points, which are extracted and matched between image frames to compute the camera’s motion. While indirect methods are typically more robust to varying environmental conditions, they may suffer from a lack of sufficient feature points in texture less or low-contrast areas. SVO utilizes a probabilistic depth filter on 2D detected features to obtain 3D features. Additionally, SVO employs bundle adjustment to optimize the position and structure of the environment. The algorithm can operate at 55 fps on small platforms and 300 fps on a laptop.

- DSO

Direct sparse odometry (DSO) was proposed in 2018 and focused on accounting for exposure time, lens vignetting, and non-linear response functions [80]. DSO is considered a visual odometry method because it does not perform global map optimization. The DSO relies on a direct photometric method that suppresses accumulative errors as much as possible instead of extracting feature points. Additionally, it splits the image into blocks and selects high-intensity pixels from regions. It then uses regions with intensity gradients for feature tracking and map reconstruction.

#### 3.1.3. RGB-D Methods

**Figure 9:**
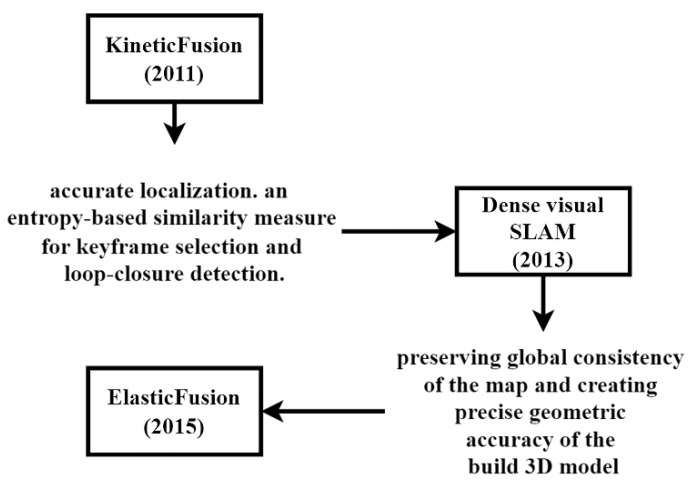
Evolution of RGB-D methods.

RGB-D cameras facilitate the perception of an environment by providing both color and depth information for each pixel [136]. These cameras employ various methods to ascertain depth, such as projecting a consistent speckle pattern onto a stereo camera or utilizing a time-of-flight sensor to gather depth information, thereby offering real-time 3D data [137]. The advent of cost-effective and compact RGB-D cameras has significantly broadened their usage in visual SLAM applications. However, it is important to note that not all RGB-D cameras rely on speckle pattern projection or time-of-flight sensors; some utilize stereo vision techniques that exploit the disparity between the perspectives of two cameras. The following presents a further discussion of visual SLAM algorithms specifically tailored for RGB-D cameras. Figure 9 shows the evolution of RGB-D methods.

A significant drawback of using RGB-D methods is the requirement for high computational resources. However, reference [138] presented an object-level RGB-D method called SLAM++ to reduce the computational cost. In this approach, 3D objects are registered in a database and replaced by 3D points whenever they are recognized during the online process.

- Kinetic Fusion

Kinect Fusion was first introduced in 2011 [103]. This method fuses depth data from a Kinect sensor into a real-time, implicit global model of the observed scene. This method builds this model by continuously fusing depth data from the Kinect sensor into a volumetric representation of the environment. It uses a voxel-based data structure to represent the 3D space, enabling efficient updates and queries. The ICP algorithm aids in aligning the depth maps with the global model, ensuring consistency and accuracy in the reconstruction process. In particular, it uses voxels to represent the 3D structure of its environment. Kinect Fusion reconstructs the 3D structure of the environment by merging depth maps in space and employs the ICP algorithm to estimate camera motion. It also generates large-scale, metrically consistent maps by integrating localization results with ICP, loop detection methods, and map optimization techniques.

- Dense visual SLAM

Dense visual SLAM was introduced in 2013 and focused on accurate localization by taking advantage of dense maps [139]. It also proposed an entropy-based similarity measure for keyframe selection and loop closure detection.

- Elastic Fusion

In 2015, Elastic Fusion was introduced as a real-time dense SLAM system designed to achieve global consistency in map creation without relying on pose graph optimization or post-processing steps [140]. This approach utilizes dense frame-to-model camera tracking and windowed surfel-based fusion, enhanced by a deformation graph, which enables continuous non-rigid surface deformations of the model. The deformation graph allows Elastic Fusion to refine the 3D map dynamically, accommodating corrections in areas with accumulated drift or distortion. As a result, Elastic Fusion attains high geometric accuracy and consistency in the reconstructed 3D environment, even without traditional optimization techniques.

### 3.2. Visual-Inertial SLAM

Visual-inertial methods integrate camera and IMU data for simultaneous localization and mapping. With the rapid development and widespread availability of camera and inertial sensors, these techniques have significantly advanced in both industrial and academic research. Moreover, AR applications have garnered much interest in visual-inertial methods. Another crucial factor to consider is the low power consumption and lightweight nature of these sensors, making them ideal for small mobile platforms.

**Figure 10:**
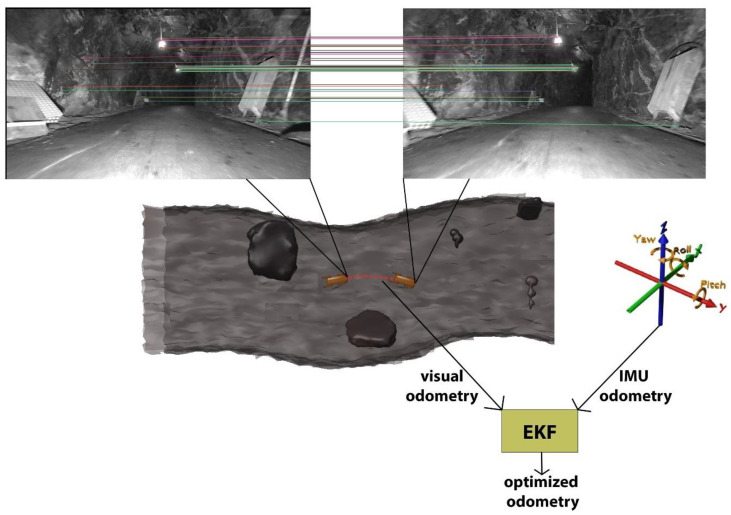
Visual-inertial SLAM workflow demonstration.

This study categorizes visual-inertial methods into two categories based on sensor fusion: loosely coupled and tightly coupled methods. A comprehensive overview of the workflow for visual-inertial algorithms can be seen in Figure 10.

#### 3.2.1. Loosely Coupled Methods

**Figure 11:**
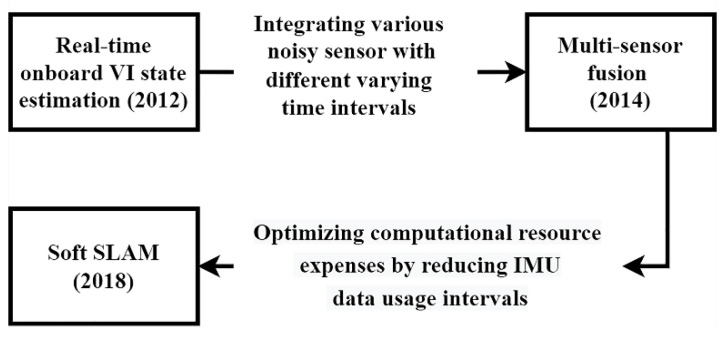
Evolution of loosely coupled methods.

The sections below outline the development of loosely coupled visual-inertial techniques. Figure 11 provides a summary of this discussion.

- Real-time onboard VI state estimation

The first real-time visual-inertial algorithm, developed in 2012, utilized onboard visual-inertial state estimation by combining a camera and an IMU for odometry [115]. This method employed consecutive camera frames to estimate the platform’s speed. The resulting data, when combined with IMU information, was fed into an EKF to estimate the state of the moving platform.

- Multi-sensor fusion

A more comprehensive study published in 2014 [141] explored the integration of various noisy sensors with differing time intervals. The research involved an IMU, a 2D laser scanner, stereo cameras, a pressure altimeter, a magnetometer, and a GPS receiver, all mounted on a small moving platform. The key features of this approach included the use of an UKF to address challenges arising from the semi-definiteness of the covariance matrix, stochastic cloning [142] for combining sensor measurements, and a filtering framework.

- SOFT-SLAM

The stereo odometry feature tracking (SOFT)-SLAM is a loosely coupled method that integrates sensor outputs to generate a dense map of the environment. First proposed in 2018 [143], the primary objective of SOFT-SLAM is to optimize computational costs by decreasing the intervals at which IMU data are used.

#### 3.2.2. Tightly Coupled Methods

**Figure 12:**
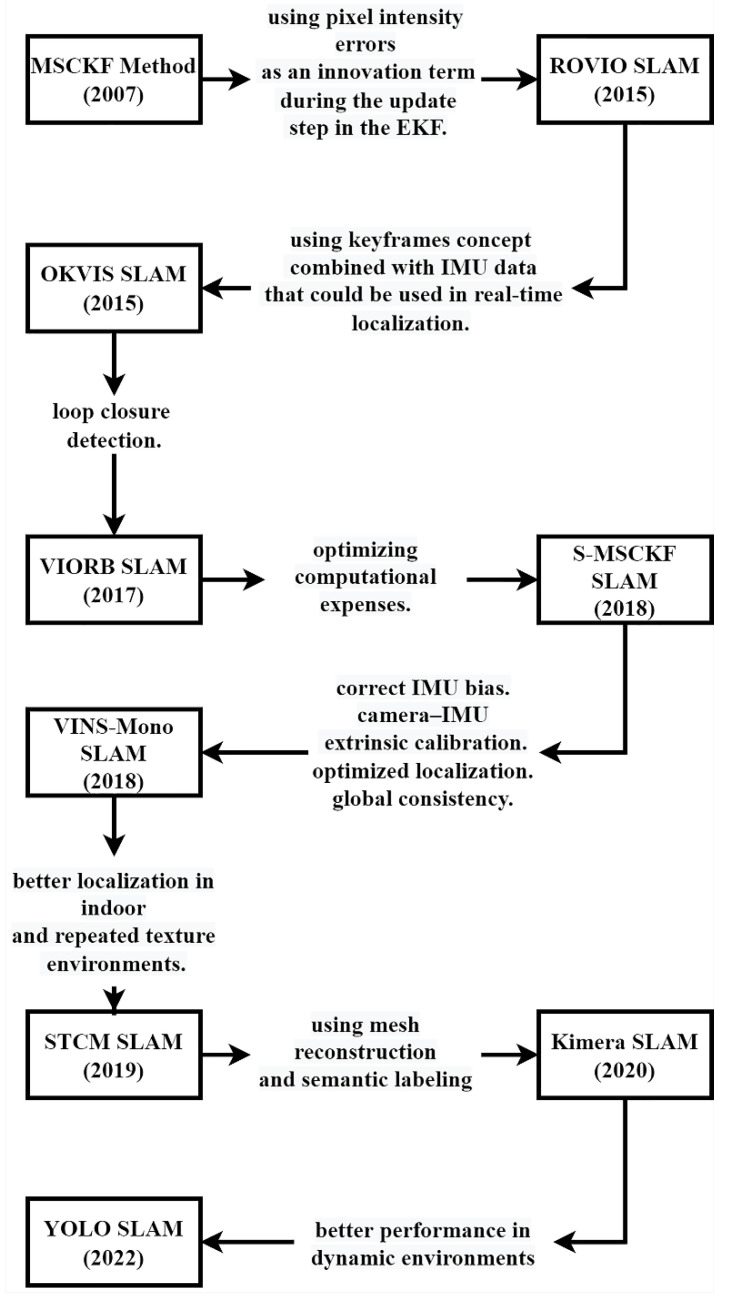
Evolution of tightly coupled methods.

The subsequent sections offer an in-depth overview of the available tightly coupled visual-inertial methods. A summary of this section can be found in Figure 12.

- MSCKF

The first algorithm that combined raw inertial sensor data and visual information for localization was introduced in 2007 [77]. The multi-state constraint Kalman filter (MSCKF) is a filtering-based method that emphasizes defining geometric constraints for a single static feature observed from multiple perspectives. Another significant aspect of the study involves utilizing the detected features without including their 3D positions in a filter state vector.

- ROVIO

In 2015, another filtering method called robust visual-inertial odometry (ROVIO) was introduced, which combines an EKF with inertial sensor data [144]. The algorithm incorporates pixel intensity errors as an innovation term during the update step of the EKF. It tracks both the 3D position of landmarks and image patch features. Furthermore, it represents feature points using a bearing vector and distance variable.

- OKVIS

In 2015, a keyframe-based visual-inertial odometry method called open keyframe-based visual-inertial SLAM (OKVIS) was proposed [145]. This optimization-based technique aimed to incorporate the concept of keyframes with IMU data. As a result, it enabled real-time localization and robust keyframe matching using a hand-held camera.

- VIORB

Previous methods did not include a loop closure feature. In 2017, a novel algorithm was introduced in the visual-inertial monocular SLAM with map reuse (named visual-inertial ORB SLAM (VIORB)) that could address accumulated drift by implementing a loop closure algorithm [129]. Additionally, the paper presented a new method for initializing the IMU unit and calculating the related bias for odometry instruments within just a few seconds.

- S-MSCKF

The original MSCKF had significant computational demands. To tackle this problem, the stereo multi-state constraint Kalman filter (S-MSCKF), a filtering-based approach, was introduced in 2018 [79].

- VINS-Mono

In 2018, another optimization-based method was introduced, which could meet various requirements for SLAM operations [146].

The visual-inertial system (VINS) can correct IMU bias measurements and calibrate camera-IMU extrinsic parameters. Additionally, it offers a loop closure detection algorithm for enhanced localization. The system is also equipped with a 4-degree of freedom (DOF) pose graph optimization to maintain global consistency.

The global pose optimization of the VINS-Mono optimizes all graphs of sequential edges (IMU measurements, vision odometry, etc.) and loop closure edges as follows.

(11) $$min_{P,\psi }(\sum_{\left(i,j\right)\in S}\left({\left|\left|r_{i,j}\right|\right|}^{2}\right)+\sum_{\left(i,j\right)\in L}\left(\rho \left({\left|\left|r_{i,j}\right|\right|}^{2}\right)\right))$$

where S and L are the sets of all graphs of sequential edges and loop closure edges, respectively. ψ represents yaw angle, P is relative position, r is the residual of the edge between frames i and j, and ρ is the Huber norm [147].

- STCM-SLAM

In EGD environments, localization can be difficult due to the presence of recurring textured features, narrow moving spaces, and loss of features. To improve localization results, a sequence of circle matching for space–time circle matching (STCM) SLAM was proposed in 2019 [148]. This approach employs a single-circle feature-matching technique and utilizes both forward and backward optical flow to track image features. Moreover, the algorithm incorporates a marginalization method to maintain keyframe constraints.

- Kimera

In 2020, Kimera was introduced as the first SLAM method integrating mesh reconstruction with semantic labeling in SLAM [149]. The algorithm comprises four main modules: visual odometry, robust pose graph optimization, lightweight 3D mesh reconstruction, and dense 3D metric-semantic reconstruction. Kimera’s visual odometry module utilizes IMU pre-integration, allowing it to fuse inertial measurements with visual data to improve robustness and accuracy, particularly in dynamic and challenging environments. The mesh reconstruction and metric-semantic modules create a detailed map of the environment and enhance it with semantic labels that offer a comprehensive understanding of the surroundings. This combination of accurate localization, mesh generation, and semantic enrichment makes Kimera a versatile SLAM solution capable of producing both metric and semantic maps.

- YOLO-SLAM

Previously, labeling-based SLAM methods failed to deliver satisfactory performance in combining object detection models to label objects in dynamic environments. In 2022, you-only-look-once (YOLO)-SLAM was introduced to tackle this issue [150]. The approach builds on ORB-SLAM 2 and incorporates a lightweight object detection network called Darknet19-YOLOv3 to label objects in the surroundings. Additionally, the method utilizes a geometric constraint technique using RANSAC to filter dynamic objects from the scene and detect features from static objects in the environment.

- ORB-SLAM3

ORB-SLAM3 is the latest iteration in the ORB-SLAM series, building upon the foundations laid by its predecessors, ORB-SLAM and ORB-SLAM2 [129]. It maintains the robust feature-based monocular SLAM approach of the original ORB-SLAM. Unlike ORB-SLAM, which was limited to monocular systems, and ORB-SLAM2, which extended support to stereo and RGB-D cameras, ORB-SLAM3 further expands its versatility by integrating multi-map support, enabling it to operate across multiple disconnected environments. Additionally, ORB-SLAM3 adds visual-inertial SLAM, which uses inertial measurement units (IMUs) to make it more accurate and reliable in tough situations where visual information is limited. This enhancement addresses one of the primary limitations of ORB-SLAM and ORB-SLAM2, which relied solely on visual data, making ORB-SLAM3 more versatile and reliable in diverse real-world applications.

### 3.3. Learning-Based SLAM

Learning-based approaches to SLAM have recently gained traction for their ability to complement or replace classic geometry-based pipelines. By leveraging deep neural networks for feature extraction, scene understanding, and pose estimation, these methods address the inherent limitations of traditional, geometry-based feature detection solutions. They exemplify a paradigm shift toward data-driven SLAM, promising improved scalability and resilience in complex real-world scenarios. This section discusses some of the famous approaches in this rapidly evolving domain, while readers can find a detailed review of NeRF-based SLAM in reference [151].

#### 3.3.1. NICE-SLAM

The method introduces a dense SLAM that combines neural implicit representations with a hierarchical grid-based encoding for scalable and detailed scene reconstruction [152]. The method optimizes multi-resolution voxel grids for geometry and color features that leverage pre-trained MLP decoders to improve reconstruction accuracy and scalability. By integrating coarse-to-fine updates, NICE-SLAM addresses limitations such as over-smoothed reconstructions and global updates that enable real-time tracking and mapping in large-scale scenes. Experimental results demonstrate its robustness and efficiency in handling diverse datasets, which outperformed existing methods in both reconstruction quality and computational efficiency. Limitations include a lack of loop closures and predictive capabilities constrained by coarse-level priors.

#### 3.3.2. NGEL-SLAM

The method combines traditional feature-based tracking, as seen in ORB-SLAM3, with neural implicit representations to achieve globally consistent and low-latency SLAM [153]. The system incorporates three parallel processes: real-time tracking, dynamic mapping, and loop closing, which ensures robust camera pose estimation and efficient map updates. Utilizing a sparse octree structure for multi-level feature storage, the mapping module enables fast convergence and memory-efficient scene representations. The introduction of uncertainty-based image rendering further enhances accuracy by selecting optimal sub-maps.

#### 3.3.3. MASt3R-SLAM

The method introduces a real-time dense SLAM framework that leverages two-view 3D reconstruction prior to achieving globally consistent poses and dense geometry from monocular inputs [154]. The system integrates point map matching, tracking, local fusion, and loop closure within a modular architecture. It handles generic, time-varying camera models without requiring known calibration, enabling robust operation in diverse and challenging scenarios. Advanced backend optimization ensures large-scale consistency, while the use of 3D priors enhances reconstruction accuracy.

#### 3.3.4. MONO-GS

Gaussian Splatting SLAM introduces a novel SLAM system that leverages 3D Gaussian splatting as its sole representation for tracking, mapping, and rendering [155]. This method enables real-time operation (3fps) with high fidelity, seamlessly unifying geometry and appearance in a memory-efficient manner. Unlike traditional representations, the system employs differentiable rasterization to refine camera poses and optimize the 3D Gaussian parameters, achieving robust tracking and dense mapping without relying on pre-trained priors or depth sensors. Evaluated on synthetic and real-world datasets, the system demonstrates superior trajectory accuracy and rendering quality, particularly in handling fine structures and transparent objects. Its isotropic regularization ensures geometric consistency, while innovations like Gaussian insertion and pruning enhance scalability and robustness for large-scale scenes.

## 4. LiDAR-Based SLAM

This section covers LiDAR-only and LiDAR-inertial SLAMs. Many LiDAR-based methods are advancements over older versions and, therefore, share similar modules. Based on their similarities, they chronologically appear after each other in the following sub-sections.

### 4.1. LiDAR SLAM

**Figure 13:**
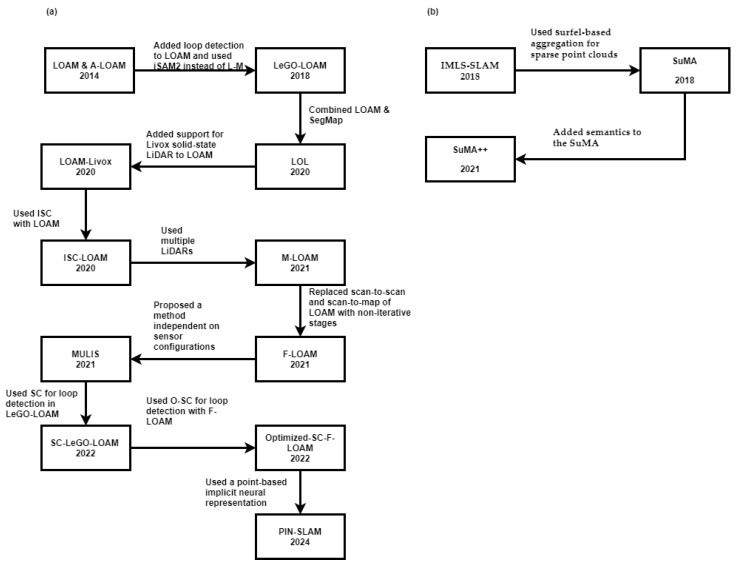
Evolution of (**a**) feature-based and (**b**) direct-based LiDAR SLAM methods.

This subsection describes algorithms that can run using either one LiDAR or multiple LiDARs. This paper divides them into feature-based and registration-based. The evolution of LiDAR SLAM is shown in Figure 13.

#### 4.1.1. Feature-Based Methods

Feature-based LiDAR methods extract and track features of a LiDAR point cloud for pose estimation. This section describes such methods.

- LOAM

LiDAR odometry and mapping (LOAM) divides SLAM into high-frequency (10 Hz) odometry for velocity estimation and low-frequency (1 Hz) odometry for mapping. The algorithm, which was proposed in 2014, only relies on LiDAR data and performs odometry by tracking the relative motion of the points included in the point cloud [156]. In the LOAM, sharp edges and patches of planar surface are selected as feature points. Instead of all points, the LOAM matches the geometrical features of consecutive scans. As a result, it achieves real-time performance.

There are some assumptions in the LOAM: (a) LiDAR is pre-calibrated, (b) LiDAR angular and linear velocities are smooth and continuous over time, (c) each scan coverage by a 2D LiDAR is defined as a sweep, (d) subscription k, $k\;\in Z^{+}$, is used to indicate the sweeps and $P_{k}$ to indicate the point cloud perceived during sweep k, $\left(\left(-\pi /2\leq \;sweep\;k\leq +\pi /2\right)\right)$3D LiDAR output is directly called $P_{k},$ (f) the right-handed Cartesian coordinate system of the LiDAR and world are called L and W, (g) in the initial position, W coincidences with L, (h) for a point $p_{i}\;\in \;P_{k}$ the coordinates are given by $X_{\left(k,i\right)}^{L}\in L$ and $X_{\left(k,i\right)}^{W}\in W_{k}$, respectively.

To solve the SLAM problem, the LOAM determines the 3D motion of the LiDAR within its environment during each sweep k. The process is conducted based on the sequence of the point cloud $P_{k}$, $k\;\in \;Z^{+}$ Then, it builds a map with $P_{k}$ for the traversed space.

Within the LOAM algorithm, after receiving data from a 2D LiDAR in each sweep, they are registered in L to form a 3D point cloud $P_{k}$. Therefore, the first two blocks can be replaced by a single 3D LiDAR block. The next module takes $P_{k}$ as its input and estimates LiDAR motion during two consecutive scans (10 Hz transform update). It relies on scan-to-scan matching, which is an iterative process. It also corrects the point cloud distortion (at 1 Hz) based on the estimated motion. Afterward, the LiDAR mapping module processes the LiDAR odometry block’s results to iteratively match and register the undistorted point cloud onto a global map (scan-to-map matching). Then, it updates the transform at 1 Hz. Finally, the last module integrates two transform updates into one 10 Hz transform update. Further details are as follows.

In the matching process, the LOAM uses feature points, which are extracted using Equation (12). Given a set S of consecutive points $p_{i}\in P_{k}$ in each scan, the smoothness of the local surface is calculated by:

(12) $$c=\frac{1}{\left|S\right|.\left|\left|X_{k,i}^{L}\right|\right|}.\left|\left|\sum_{j\in S}\left(X_{\left(k,i\right)}^{L}-X_{\left(k,j\right)}^{L}\right)\right|\right|\;,\;j\neq i$$

Calculated c values sort points in a scan. Next, edge and planar points are recognized based on the maximum and minimum c values, respectively. Note that a point $p_{i}$ is only selectable as a feature point if its corresponding crossing surface is not approximately parallel to the LiDAR beam. In addition, S must not contain any points that are disconnected from $p_{i}$ in the direction of the LiDAR beam while simultaneously being closer to the LiDAR. Finally, points located on the border of an occluded object are not considered.

First, the LOAM’s odometry module re-projects all $P_{k}$ from the sweep k-start time $t_{k}$ to ${\bar{P}}_{k}$ at timestamp $t_{k+1}$ (i.e., sweep k-end time). It considers LiDAR’s motion using ${\bar{P}}_{k}$ and $P_{k+1}$ during each sweep, hence ${\bar{P}}_{k+1}$ is called an ‘undistorted point cloud’. Then, the odometry module calculates the pose transformation matrix between the time t and $t_{k+1}$ at the end of each sweep. Assume that $\varepsilon_{k+1}$ and $h_{k+1}$ are sets of edge points and planar points, respectively. Additionally, consider $d_{\varepsilon }$ as point-to-line distance and $d_{h}$ as point-to-plane distance, which are geometrically obtained from LiDAR measurements. Now, it will be possible to find a relationship $f_{s}$ between the edge points and their corresponding edge lines by Equation (13). Similarly, Equation (14) defines a relationship $f_{h}$ between the planar points and their corresponding planar patches.

(13) $$f_{\varepsilon }\left(X_{\left(k+1,i\right)}^{L},\;T_{k+1}^{L}\right)=d_{\varepsilon },\;i\in \varepsilon_{k+1}$$

(14) $$f_{h}\left(X_{\left(k+1,i\right)}^{L},T_{k+1}^{L}\right)=d_{h},\;i\in h_{k+1}$$

where $T_{k+1}^{L}$, which is a 6×1 vector of translations and rotations, is defined to be the LiDAR pose transformation matrix between the time t and $t_{k+1}$ in L. To calculate the LiDAR motion, the LOAM uses the Levenberg–Marquardt (L-M) method by a nonlinear iterative approach (Equation (15)) replacing $T_{k+1}^{L}$ with the calculated values.

(15) $$T_{k+1}^{L}-{\left(J^{T}J+\lambda diag\left(J^{T}J\right)\right)}^{-1}J^{T}d\;J=\frac{df}{dT_{k+1}^{L}},\;\;f\left(T_{k+1}^{L}\right)=d$$

In the above equation, λ is a factor L-M obtains. f is a nonlinear function that stacks Equations (13) and (14), containing all feature points. Lastly, d denotes the corresponding distances of the feature points.

The LOAM’s mapping module is based on the same principles used in the odometry module but takes ${\bar{P}}_{k+1}$ and $T_{k+1}^{L}$. If $T_{k}^{W}$ is the LiDAR pose transformation matrix with the shape of 6×1 at the time $t_{k+1}$ in the coordinate system of the world $\left(W\right)$, the module extends $T_{k}^{W}$ between the time $t_{k+1}$ and $t_{k+2}$ (1 Hz transform update). This is performed by accumulating $T_{k+1}^{L}$ values. The transform integration module then sequentially integrates $T_{k+1}^{L}$ and $T_{k}^{W}$ to obtain the output transform at 10 Hz. The mapping module also projects the ${\bar{P}}_{k+1}$ into W and calls it ${\bar{Q}}_{k+1}$. Finally, it matches ${\bar{Q}}_{k+1}$ and the accumulated point cloud (map) until sweep k. The matching process is based on the L-M optimization of $T_{k}^{W}$ to update and register the undistorted point onto the map at 1 Hz.

The LOAM revolutionized LiDAR-based SLAM. It is the foundation of many other algorithms that have either improved or used some of its modules. However, it has some drawbacks. As its main drawback, the LOAM does not correct the translation errors because it saves data in a voxel-global map. A voxel map uses voxels to save the map of the 3D structures. A voxel map compounds the incorporation of other data sources, such as wheel odometry and loop closure because the LOAM’s optimizer is inefficient when the voxel-global map becomes dense. The other issue appears in large-scale tests where the LOAM suffers from accumulated translation and drift errors. It happens as the LOAM only relies on the scan-matching method [157]. As a result of accumulating the map and the output transform, excessive memory consumption and potential overview are expected in long runs of LOAM. Lastly, the LOAM’s complicated source code negatively affects its run time despite its ability to achieve real-time performance. To address the last problem, an advanced implementation of LOAM (A-LOAM) optimized the alignment between two scans using the Ceres-solver factor graph optimization library. It also simplified the LOAM code structures by modifying its mathematical derivations and removing redundant operations. For example, in the ‘laserOdometry.cpp’ file, it used a high-level C++ library (Eigen) within the ‘TransformToStart’ part to remove many lines.

- LeGO-LOAM

To reduce the LOAM’s drift errors, lightweight and ground-optimized LiDAR odometry and mapping (LeGO-LOAM) added loop detection to the LOAM in 2018. The LeGO-LOAM relies on Euclidean distance-based ICP. It aims to be more accurate than the original LOAM and use fewer computational resources [158].

In the LeGO-LOAM, first, the segmentation module receives the 3D point cloud of each scan and projects it onto a range image. A range image contains a range value and other features, such as LiDAR returning beam strength (intensity), for each pixel. The outputs of the segmentation module are points that represent large objects as well as ground points, hence, the adjective ground optimized. Next, the feature extraction module takes the segmented points and extracts them like the LOAM. The adopted modules from the LOAM are represented by blocks with gray backgrounds and dotted borders hereafter. By using the extracted features, LiDAR odometry calculates the LiDAR motion through a two-step L-M method that reduces the run time, hence the lightweight adjective in LeGO-LOAM’s name [158]. Step one takes planner features and estimates perpendicular transformation, roll, and pitch. In the next step, edge features are used to estimate the planar transformation and yaw. During the second step, the first step’s results are the constraints. The LiDAR mapping module registers feature points to a global map through three consecutive submodules, namely feature matching, Euclidean-based ICP loop detection, and incremental smoothing and mapping 2 (iSAM2) optimization. The module saves planner and edge feature sets rather than a single point-cloud map in the LOAM. Moreover, it uses a K-D tree data structure compared to the voxel-global map of the LOAM. Finally, the transform integration module of the LeGO-LOAM works similarly to the LOAM.

The iSAM2 incrementally updates the solution in real-time as new data arrives, making it computationally efficient and suitable for dynamic environments. In contrast, L-M processes all data in a batch to find the optimal solution, which is more accurate but computationally intensive. Therefore, iSAM2 is ideal for real-time applications, while L-M is better suited for scenarios requiring high accuracy without real-time constraints.

- LOAM-Livox

LiDARs come in two types: solid state and mechanical. Solid-state LiDARs are free of mechanical mechanisms and, therefore, less prone to failures. However, they have a limited field-of-view (FoV) and irregular scan patterns. To use solid-state LiDARs (especially Livox LiDARs), the LOAM-Livox (2020) directly matches new frames with a global map for pose estimation, as there is no sweep-time lag [159]. Then, the pose estimation output assists in the registration of a new frame to the global map. Consequently, mapping and pose estimation have the same 20 Hz rate. Its feature extraction module is also like the LOAM, but it takes the intensity into account. Based on the intensity, it selects so-called good points (i.e., points with higher intensity values) and detects the edge features. Moreover, it only keeps feature points in the system memory and saves raw points in the hard drive. Raw points are then used for offline global optimization if required. Thus, the LOAM-Livox is significantly faster than the original LOAM.

- LOL (a combination of SegMap and LOAM)

SegMap stands for segment-based mapping. This algorithm extracts segments from 3D point clouds for localization and mapping. It reduces sensitivity to viewpoint and local structural changes. As a result, it processes large-scale 3D data faster [160]. In 2020, LiDAR-only odometry and localization (LOL) combined the LOAM and segment-based mapping (SegMap) to compensate for the drift error [161]. To this end, it finds the geometrical similarities of the locations between an online map and an offline map. The online map refers to the map generated during the moving platform traverse, while the offline map is a prior map of the environment. Thus, LOL works best when an offline map exists, such as underground sites where there is usually a prior map.

LOL starts by taking an offline point cloud map. Then, the offline segmentation module accumulates the point cloud map in a dynamic voxel grid and extracts segments. The segment extraction method includes two algorithms. The first one removes ground points and clusters the remaining points based on their Euclidean distances. Next, the second algorithm extracts planar surfaces by computing the local normal and curvature of each point. Using the segmented point cloud, a convolutional neural network (CNN) descriptor extracts features such as large objects. Due to the segmentation and descriptor extraction, a segment database is created.

Afterward, the LOAM algorithm takes the 3D LiDAR point cloud and calculates the pose transforms and pose estimations. Once the pose estimation is available, the 3D LiDAR point cloud is also used to build a local map. It is followed by an online segmentation and descriptor extraction with the same principles as the offline modules. Further, the matching module searches the prior map and local map segmented databases. The searching module works based on the global position of the segment centroids and compares included features to find corresponding candidates.

To remove the false matches, the verification module applies a two-stage RANSAC-based method before further refining previous matching results by an ICP method. Stage one searches the segment databases in a limited range between the odometry results and the centroids to find matches. Then, it checks the found matches by criteria such as the maximum absolute difference of translation vectors between the matching pair centroids. Stage two takes the remaining matching segment centroids and aligns them using RANSAC, iteratively. If it converges, it keeps the matching segment centroids.

Finally, the incremental pose graph mapping module takes ICP refinement and the LOAM outputs to update the trajectory. It also sends feedback on the local map segmentation by global pose estimation.

- ISC-LOAM

LeGO-LOAM had a loop detection module. However, it was sensitive to changes in the LiDAR viewpoint because it would compare the current pose with previously viewed poses from a certain point. As a result, loop detection would fail in situations like revisiting a place reversely or revisiting a corner. To solve the problem, a place descriptor called scan context (SC) was created in 2018. It describes a place based on structural information [162]. The SC records a 3D structure from a LiDAR view without prior training or using histograms (such as reference [163]). However, SC does not take intensity into account. Thus, the intensity scan context (ISC) was proposed in 2020. It aimed to consider both geometry and intensity characteristics [164]. It calculates the surface reflectance structure based on the intensity reading to reduce the illumination variation problem. Subsequently, it improves the loop detection performance. In terms of formulation, SC and ISC are remarkably similar, but the former only uses a geometrical structure.

- F-LOAM

As the name suggests, the fast LOAM (F-LOAM) is an even faster version of the LOAM compared to the A-LOAM in 2021. The F-LOAM is like the LOAM in that it compensates for distortions using features included in the point cloud [165]. Instead of scan-to-scan and scan-to-map processes used in the LOAM, the F-LOAM proposed two non-iterative stages to increase computational efficiency. However, it still performs pose optimization in an iterative manner.

- MULLS

Most LiDAR methods in the current paper represent point clouds depending on the sensor configurations. For example, the LOAM uses ring representation while LeGO-LOAM, surfel-based mapping (SuMA), and SuMA++ use range image representation (SuMA and SuMA++ are described Section 4.1.2). Thus, it is necessary to specify the parameters of the LiDAR model, including the field of view and scan-line distribution. By contrast, versatile LiDAR SLAM via multi-metric linear least squares (MULLS) proposed taking sets of 3D coordinates as continuous inputs in 2021. Consequently, the MULLS maintains 3D structure information and is adaptive to several types of LiDARs [104].

The front end of the MULLS includes geometric feature points extraction and encoding (GFPEAE), multi-metric linear least square ICP (MULLS ICP), and map management modules. The GFPEAE has three submodules, including the ground filter, the nonground points classification based on principal component analysis (PCA), and the neighborhood category context (NCC) encoding. The ground filter submodule divides the point cloud into equal 2D grids once it has projected them onto a reference plane. Next, a dual threshold filter first compares the minimum point heights of grids and neighboring grids with a threshold. Then, it compares the difference between the heights of points and with another threshold.

The nonground points classification submodule works in parallel with the ground filter, which is based on PCA. After downsampling nonground points to a fixed number of points, PCA is applied to further reduce the dimension of data. Then, the covariance of the nearest point within a certain sphere radius is calculated. Next, the eigenvalue decomposition determines the eigenvalues and the corresponding eigenvectors. The magnitude of the local feature (which are linearity, planarity, and curvature) and the directions of the first and third eigenvectors generate five categories of features, including facade, roof, pillar, beam, and vertex. Then, NCC describes each vertex key point approximately without any additional computations. Note that some of these features do not apply in EGD environments such as facades and roofs.

MULLS ICP and map management modules work together. Based on the NCC description, a scan-to-scan MULLS ICP estimates the 3D motion of the LiDAR within its environment using static points in a local map. Then, scan-to-map MULLS ICP takes the estimated transformation matrix as initial values and feature points as targets until convergence. Next, feature points are added to the local map after filtering dynamic points. Finally, the back end of MULLS constructs loop closure constraints using a map-to-map global registration. It is based on two algorithms: (a) truncated least squares estimation and semidefinite relaxation (TEASER) [166] and (b) a submap-based pose graph optimization (PGO).

- M-LOAM

Using multiple LiDARs benefits maximizing the environmental perception of the moving platforms. To this end, multi-LiDAR odometry and mapping (M-LOAM) proposed simultaneous extrinsic parameters calibration, odometry, and mapping for more than one LiDAR [167]. M-LOAM, which was proposed in 2021, starts with edge and planar features extraction. Once motion estimation and extrinsic parameters are initialized, a sliding window-based odometry estimates the pose of the multi-LiDAR system. Its odometry module includes online calibration and extrinsic parameters convergence monitoring. At the final stage, the mapping module builds a global map, optimizes poses, and reduces data uncertainty.

- SC-LeGO-LOAM

It is effortless to integrate scan context with the other algorithms. In 2022, SC-LeGO-LOAM improved the LeGO-LOAM by implementing the SC algorithm for loopback detection. It reduced the point cloud dimensions, which enhanced computational efficiency [15]. The SC-LeGO-LOAM is like LeGO-LOAM. Thus, we only focus on the difference. The adopted modules from the LeGO-LOAM are represented by gray blocks with dashed borders hereafter.

The LiDAR mapping module takes the raw point cloud in addition to the LiDAR odometry results for generating a global map. Within the module, the feature matching block is directly connected to the iSAM2 optimization block. Moreover, there is a new loop detection submodule that sends its output to the ICP block. The submodule has four blocks. The descriptor block divides the 3D point cloud into sectors and rings. The next block generates an SC and sends it to the following block. The nearest neighbor search block finds the geometry similarity between the candidate and the SC query. Finally, the fourth block uses the output of the previous block to calculate the similarity score.

SC integration aims to be resilient to view changes. Therefore, SC-LeGO-LOAM has the potential to achieve better pose estimation accuracy in EGD environments, which makes it a candidate for further investigation in this paper. This is because of the feature-poor nature of such environments where changing the view does not provide extra information and compounds the pose estimation task.

- Optimized-SC-F-LOAM

Optimized-SC-F-LOAM (2022) is like SC-LeGO-LOAM in that both improve their successor by SC integration [101]. However, the former uses an optimized version of the SC and a feature-based matching (instead of the ICP) within its loop closure module.

By using a fixed distance threshold, the original SC method can determine and exclude point-cloud pairs that are highly similar yet far away. This method may induce false detection depending on how far the fixed threshold is set. To solve the problem, the loop closure module of the optimized-SC-F-LOAM relies on an adaptive distance threshold to improve loop closure detection. The use of adaptive thresholds and removing ICP matching within the loop closure detection process has made the optimized-SC-F-LOAM more accurate and computationally efficient than the original F-LOAM.

- PIN-SLAM

Point-based implicit neural (PIN)-SLAM is a SLAM system designed to achieve global map consistency using a point-based implicit neural representation, hence the name [168]. This system leverages an elastic and compact neural map that alternates between incremental learning of the local implicit signed distance field and pose estimation. By using sparse optimizable neural points, PIN-SLAM can obtain robust and accurate localization even in challenging environments.

Although the current version of PIN-SLAM only relies on LiDAR, the system can handle various range sensors, including RGB and RGB-D cameras. The use of voxel hashing for efficient neural point indexing and fast implicit map-based registration allows PIN-SLAM to operate at the sensor frame rate on a graphics processing unit (GPU). This approach enhances pose estimation accuracy and supports loop closure detection, confirming a global map that can be reconstructed as meshes.

#### 4.1.2. Direct Methods

This section describes methods that directly register LiDAR point cloud onto a map and use all points for pose estimation.

- IMLS-SLAM

In 2018, IMLS-SLAM proposed an implicit moving least squares (IMLS) surface matching framework [169]. IMLS is a low-drift alternative algorithm to the ICP algorithm. It gives the distance approximation of any point x in three-dimensional space to the point cloud ($P_{k}$) implicit surface I by:

(16) $$I^{P_{k}}\left(x\right)=\frac{\sum_{p_{i}\in P_{k}}W_{i}\left(x\right)\left(\left(x-p_{i}\right).n_{i}\right)}{\sum_{p_{i}\in P_{k}}W_{i}\left(x\right)}$$

where $W\left(x\right)=e^{{\left|\left|x-p_{i}\right|\right|}^{2}/h^{2}}$, n is normal at point $p_{i}$, and h in W is the IMLS surface definition parameter. To find the location of the current scan (CS) in the point cloud, the IMLS-SLAM minimizes $\sum_{x_{i}\in CS}{\left|\left|I^{P_{k}}\left(Rx_{i}+t\right)\right|\right|}^{2}$ by optimizing transformation parameters (R and t). Because of exponential weights ($W\left(x\right)$), The IMLS-SLAM projects all the current scan’s points on the IMLS surface ($projected\;\;x_{i}=x_{i}-I^{P_{k}}\left(x_{i}\right)n_{i}$). Thus, its formulation makes a new K-D tree representation from all points to find the nearest neighbors. As a result, it lacks real-time performance.

- SuMA

LiDAR point clouds are usually sparse which causes drift errors in pose estimation. Aggregating the information of previous timestamp scans while scanning the environment can make a dense representation of the environment and, therefore, alleviate the problem. Surfel-based mapping (SuMA) presented such an approach for odometry and mapping in 2018 [170]. Receiving the point cloud, the pre-processing module generates a vortex map. Then, it maps a 2D image coordinate to a point to generate its corresponding normal map.

Taking the vortex map and normal map, the pose estimation module first constructs a surfel map and renders the vertex map and normal map at t−1. The surfel map contains the last point clouds up to t−1. Following that, the odometry submodule aligns the rendered vertex map and current vortex map by using ICP to estimate frame-to-model transformation from t−1 to t. Afterward, the submodule uses this transformation and updates the global pose. Finally, the surfel map is updated.

The next module takes the vortex map, normal map, transformed points from the world-to-current frame, and a so-called inactive surfel map. First, the loop closure detection module selects one loop closure candidate in the inactive surfel map based on the minimum transitional Euclidean norm. Next, it aligns the candidate with the transformed points by frame-to-model ICP. For solving small overlap scenarios, the module composes a virtual view of the map before any loop closure. Accordingly, it uses the transformation between the up-to-date point cloud and the potential loop closure to generate composed vertex and composed normal maps. After that, it renders the inactive map at the candidate position to fill composed vertex and composed normal maps. If a point in the vortex map is closer to the candidate than the composed vertex, points are added to the composed maps from the vortex map and normal map. To validate the candidate, the module uses the vortex map to find the consistency between the composed view and up-to-date inputs. Finally, the verification module verifies loop closure at time t in the subsequent timesteps $\left[t+1,\ldots ,\;t+\delta_{verification}\;\right]$ before sending its constraints to the pose graph optimization.

In general, dense-based methods like the SuMA are useful to recognize places and relocate them in a feature-poor environment such as a long tunnel. However, dense-based methods are computationally intensive due to the pixel-to-pixel matching process. Thus, they require GPU-based parallel computations [167].

- SuMA++

SuMA++ is an extension for the SuMA that added semantics to the SuMA in 2021 [171]. The semantics are extracted by FCN RangeNet++ [172]. Before running the method, its semantic segmentation networks must be tuned and trained with the desired data. Therefore, a moving platform cannot use SuMa++ with a live data stream without prior training.

### 4.2. LiDAR Inertial SLAM

This subsection describes methods that use IMU and LiDAR(s) as the main sensors. Note that some of them can also use extra inputs such as visual, wheel, and kinematic odometry sources.

**Figure 14:**
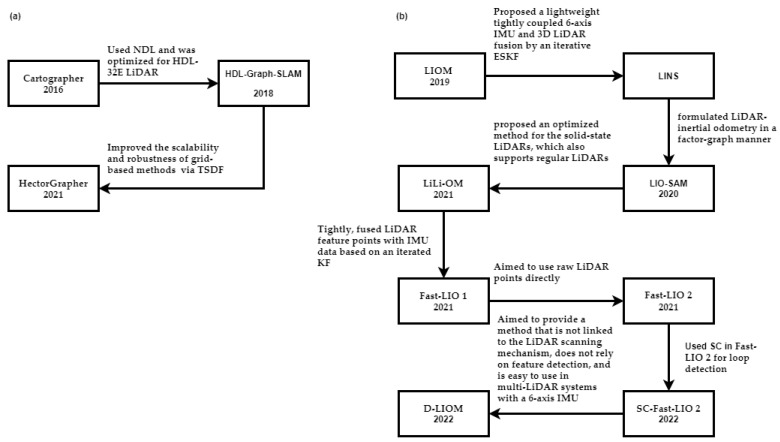
Progress of (**a**) loosely and (**b**) tightly coupled LiDAR-inertia methods.

Adding IMU can aid the LiDAR-based SLAM by fusing IMU and LiDAR data to compensate for errors, eliminate point cloud distortion, and manage probable sensor failures [173,174,175]. Like the visual-inertial SLAM, there are two types of LiDAR-inertial SLAMs, namely tightly and loosely coupled methods. Most methods in both categories are featured-based. However, there are a few exceptions. Specifically, direct LiDAR-inertial odometry and mapping (D-LIOM) [102] and fast LiDAR-inertial odometry 2 (Fast-LIO 2) [176] are direct methods in the tightly coupled category. The progress of LiDAR-inertia SLAM is shown in Figure 14.

#### 4.2.1. Loosely Coupled Methods

This section includes methods that couple LiDAR and IMU loosely, which use data from both sensors independently without feedback.

- HDL-Graph-SLAM

The high-definition LiDAR graph SLAM (HDL-Graph-SLAM) (2018) is a loosely coupled graph optimization-based method that implements normal distribution transformation (NDT) and requires the g2o optimization library, which was developed for Velodyne HDL-32E LiDAR [177]. It solves SLAM by building and optimizing a graph with nodes as parameters and edges as constraints, respectively. The supported constraints encompass GPS, gravity, orientation, loop closure, and the floor plane detected in a point cloud.

The generated map tends to bend as it gets larger because of the accumulated rotational error, which makes the system unstable. To tackle the problem, the HDL-Graph-SLAM uses GPS and floor plane constraints. It introduced GPS position-based constraints for outdoor environments where the ground is not flat and GPS is allowed. On the other hand, floor plane constraint is designed for enclosed or GPS-denied environments with a single flat floor. It first applies RANSAC to the point cloud to detect the ground plane. Every 10 s, a ground plane constraint edge is added to the graph if the normal of the detected plane is nearly vertical. Then, the pose graph is optimized so that the ground plane detected in each observation remains the same. The pose estimation is simplified in this approach by downsampling the point cloud. As a result, the HDL-Graph-SLAM is more stable at the price of accuracy.

Using IMU constraints, the HDL-Graph-SLAM integrates the NDT point cloud registration with an angular-velocity-based pose prediction method using the UKF [178] for the pose estimation process. The loop closure constraint performs loop detection, hence its name. Loop candidates are initially identified based on transitional distances (if their values < a threshold) and trajectory lengths (if their values > a threshold). NDT is then applied between the nodes of each loop candidate to validate it. Finally, the loop is added to the graph as an edge if the NDT correspondence score is lower than a threshold (e.g., 0.2).

- HectorGrapher and Cartographer

In 2016, Cartographer was introduced to provide real-time SLAM across a variety of platforms and sensors [179]. Built upon Cartographer, HectorGrapher (in 2021) aimed to improve the scalability and robustness of grid-based methods in challenging scenarios [180]. HectorGrapher works with ground-moving platforms.

First, HectorGrapher combines IMU and wheel odometry data to estimate the platform’s pose with low latency using the L-M method of the GTSAM. To ensure the precision of a high-resolution grid and the robustness of a lower-resolution grid, the LiDAR-inertial module uses a multi-resolution truncated signed distance function (TSDF) for scan-to-map registration. The TSDF is a 3D voxel array that indicates each voxel with the distance to the nearest surface. After taking the point cloud, it registers wheel-inertial odometry and LiDAR scans in a joint optimization using the L-M method of the Ceres solver. Then, The LiDAR-inertial module compensates for drift errors. In both Cartographer and HectorGrapher, the large-scale pose graph module employs a branch-and-bound method [179] to perform loop detection. The module ensures global consistency by eliminating accumulated errors.

Both HectorGrapher and Cartographer use LiDAR measurements directly. Thus, they are compatible with various LiDAR configurations. However, they couple different sensor measurements loosely that may affect the back-end performance. Compared to Cartographer, HectorGrapher achieved higher localization accuracy, based on the results published by the developers, due to its multi-resolution TSDF. However, the multi-resolution TSDF compounds the optimization problem and increases computational costs.

#### 4.2.2. Tightly Coupled Methods

This section describes tightly coupled LiDAR-inertial methods, which use raw data from LiDAR and IMU sensors at the initialization step and include feedback within their structure.

- LIOM

LiDAR inertial odometry and mapping (LIOM) from 2019 is a tightly coupled LiDAR-IMU method that jointly optimizes the data of both sensors [181].

In the LIOM system, the state prediction module takes the accelerator and gyroscope data of the IMU and predicts the upcoming state. Simultaneously, the pre-integration module calculates pose changes between two consecutive timestamps based on the IMU data.

Mechanical 3D LiDARs have a circular moving mechanism to capture the point cloud. Therefore, the movement of the platform distorts the point cloud due to the trivial differences between the true position and the captured position of the points. To tackle the problem, the next module assumes a linear position change and takes the predicted state to de-skew the point cloud. Next, the de-skewed point cloud is sent to the feature extraction module, which is adapted from the LOAM. Afterward, the local map management module merges the previous extracted feature points within a local window with respect to the corresponding optimized states. The next module receives the predicted state, extracted feature points, and the local map to find the relative LiDAR measurements. Finally, the joint non-linear optimization module takes pre-integrated outputs and relative LiDAR measurements to calculate a MAP estimation. It also provides feedback on the state prediction and local map management modules.

The LIOM obtains optimal states using a fixed-lag smoother and marginalization method. It defines a cost function with the Mahala Nobis norm and minimizes it to obtain the MAP estimation. Further, the Gauss–Newton algorithm of the Ceres solver library solves the cost function in the form of a non-linear least square. Due to the simultaneous processing of all sensor measurements, the LIOM lacks real-time performance.

- LINS

Built upon LeGO-LOAM, the LiDAR-inertial state estimator (LINS) proposed a lightweight, tightly coupled 6-axis IMU and 3D LiDAR fusion by using an iterative error-state Kalman filter (ESKF) in 2019 [182]. The ESKF generates new feature correspondences in each iteration that reduce the optimization problem’s dimensions. Thus, the LINS is faster than the LIOM and achieves real-time performance.

To achieve long-term stability, the LINS adjusts the local reference frame after each LiDAR scan. Then, it updates the global pose estimation based on the estimated relative pose of two consecutive local frames.

The LINS’s structure has four modules, namely, segmentation, feature extraction, LIO, and mapping. Segmentation and feature extraction modules are directly adopted from the LeGO-LOAM. Similarly, the mapping module was built upon the LeGO-LOAM that refines initial odometry by a global map before updating the map. On the other hand, the LIO module is new and computes the initial odometry by ESKF. The module includes state propagation and state update submodules. The former propagates the error state, error-state covariance matrix, and state prior values for the ESKF process. The latter updates the state through an iterative scheme [183].

Although the LINS is fast and stable, it only relies on previous LiDAR scans at the scan matching stage, which is a sparser point cloud than a local map. A sparser point cloud induces less pose estimation and mapping accuracy in featureless environments. To solve the issue, it employs IMU data, but the moving platform must start on a flat surface with almost zero, roll, and pitch values.

- LIO-SAM

In 2020, tightly coupled LiDAR inertial odometry via smoothing and mapping (LIO-SAM) formulated LiDAR-inertial odometry in a factor-graph manner. It enabled the incorporation of relative measurement, absolute measurement, and loop closure detection from different methods as factors into the system [157].

In the LIO-SAM, first, the IMU pre-integration module calculates the IMU motions based on the reference [184]. Next, the image projection module takes the previous module’s output as well as raw IMU and LiDAR measurements. It initializes the transformation matrix and de-skews the point cloud. Afterward, the point cloud’s features are extracted using the LOAM’s extraction method. The next module defines a keyframe when the moving platform’s pose crosses a pre-defined threshold for reducing the computational cost. Following that, a sliding window approach builds a local map by a fixed number of new keyframes, so-called sub-keyframe voxel mapping, hence the name. Following that, the scan matching module finds the corresponding features between the local map and the recent LiDAR frame by the LOAM’s scan matching method before computing relative transformation. Afterward, the LIO-SAM detects loops by directly adopting LeGO-LOAM’s loop detection module. Finally, all factors are sent to the iSAM2 graph optimization, hence the term smoothing in the LIO-SAM. Note that it is possible to add other factors, such as GPS [185].

In practice, the LIO-SAM transforms the sub-keyframes into the current state frame as an alternative method and names it a real relative transformation. Additionally, the LIO-SAM is compatible with alternative methods for its scan matching module (e.g., references [89,186]) and loop closure module (e.g., reference [187] and SC).

Compared to the LIOM, the LIO-SAM achieves a much better performance in outdoor environments, based on the results published by the developers, due to its local scan matching, while it does not need a flat surface to start, unlike the LINS. However, the LIO-SAM is extremely sensitive to the sensor’s configuration and the IMU’s extrinsic parameters tuning.

- LiLi-OM

In 2021, Livox LiDAR-inertial odometry and mapping (LiLi-OM) proposed an optimized method for the solid-state LiDARs (especially for Livox Horizon LiDAR), which also supports regular LiDARs [188].

The LiLi-OM’s structure has three main modules, including pre-processing, scan registration, and back-end fusion. The pre-processing module takes and downsamples the point cloud before de-skewing it by IMU gyroscope data. Then, it extracts the planar and edge features of the de-skewed point cloud using a novel feature-extracting method. If the second largest eigenvalue is significantly (3.33 times) greater than the smallest one, points in a patch are defined as planar features. Similarly, edge features are extracted if the largest eigenvalue is 4 times larger than the second-largest eigenvalue. Next, the scan registration module takes the extracted features. It estimates poses by the scan-matching method used in the LOAM and builds a local map. Using the estimated poses, it gives feedback on the de-skewing submodule and selects keyframes. It selects a keyframe if the similarity between the current frame and the local map is less than 40% while the time difference between the current frame and the last keyframe is more than the time between two consecutive regular frames. Simultaneous with the pre-processing, the back-end module fuses LiDAR and IMU’s pre-integrated data. Before optimization, IMU pre-integration initializes all frame poses. Then, the back-end module takes keyframes and the local map to calculate the poses of the keyframes through a sliding window-based optimization. Next, it uses the two oldest keyframe poses as constraints of a local factor graph optimization to obtain the regular fame poses. Afterward, the calculated poses and keyframe features are used for building a global pose graph, which outputs a global map and serves as input to an ICP-based loop detection submodule. The loop detection submodule only uses keyframe nodes. Lastly, the global pose graph invokes detected loop closure as its constraint to update the local map.

- Fast-LIO 1

Similar to the LINS, Fast LiDAR-inertial odometry (Fast-LIO), which was proposed in 2021, tightly fused LiDAR feature points with IMU data based on an iterated KF [189].

The Fast-LIO has three main modules including pre-processing, state estimation, and mapping modules. The pre-processing module first accumulates LiDAR measurements over a certain time. Then, it extracts the point cloud’s features based on LOAM and LOAM-Livox when mechanical LiDAR and solid-state LiDAR are used, respectively. The state estimation module has three submodules. It starts with a forward propagation of the state and its covariance matrix by taking IMU data. The next submodule eliminates the relative motion of the feature points and fuses the feature points with the forward propagation’s output. Then, the iterated KF updates the state. Finally, the mapping module receives the updated feature points to build a global map and retrieves a sub-map for further residual computation.

Compared to the LINS, the KF formulation of the Fast-LIO is much simpler. Thus, it considers an average of 784 feature points per scan instead of an average of 147 points in the LINS. This allows a real-time performance in the Fast-LIO [189].

- Fast-LIO 2

Adopting the tightly coupled KF of the Fast-LIO, the second version aimed to use raw LiDAR points directly in the same year [176].

Fast-LIO 2 is remarkably like the Fast-LIO 1. In this method, many modules are adopted from the LeGO-LOAM. Therefore, we only focus on the differences. As a result of taking LiDAR data directly, Fast-LIO 2 does not have any feature extraction module and is adaptable to LiDARs with different scanning methods. Moreover, its mapping module uses an incremental K-D tree (iK-D tree) structure for global map data maintenance that provides an incremental map update and dynamic re-balancing. To remove redundant points, the mapping module deletes older timestamp points from the iK-D tree in a box-wise manner if the LiDAR FoV range intersects the map boundaries.

Fast-LIO 2 provides two main advantages. Firstly, it allows the consideration of subtle features since it is not limited to extracting features. Thus, it achieves better accuracy. Secondly, the iK-D tree naturally supports downsampling, which makes the system more computationally efficient and faster. Note that there is also an SC version of Fast-LIO 2, which aims to integrate SC-based loop detection into the system [190].

- D-LIOM

Similar to the Fast-LIO 2, tightly coupled direct LiDAR-inertial odometry and mapping (D-LIOM) does not depend on the points cloud’s features [102]. The D-LIOM (2022) aims to provide a method that is not linked to the LiDAR scanning mechanism, does not rely on feature detection, and is easy to use in multi-LiDAR systems with a 6-axis IMU.

In the D-LIOM, first, point clouds of multiple LiDARs are synchronized regarding time and space. Then, the pre-integration module takes IMU data and the primary LiDAR’s time to calculate IMU motion. Following that, LiDAR synchronization and IMU pre-integration modules send their output to the next module for de-skewing the synchronized point cloud. Afterward, the scan-to-submap matching module directly registers scans to sub-maps using NDT. After system initialization, the front-end’s direct LiDAR-inertial odometry (LIO) creates a local factor graph using scan-to-submap matching, IMU pre-integration, and gravity prior estimation constraints in the time window of the sub-map. It also gives feedback on system initialization, IMU pre-integration, and gravity prior estimation modules. Finally, the back-end module first computes a submap-to-submap loop detection and constructs a global pose graph. The module then removes accumulated errors and estimates poses based on comparisons of projected 2D sub-maps. As a result, D-LIOM can run stably in large-scale scenes.

Compared to the tightly coupled methods, the D-LIOM can achieve higher accuracy when revisiting a place [102]. The main reason is its submap-to-submap loop detection method that is more effective in a long-term run than, for example, the empirical distance threshold-based method used in the LIO-SAM.

Moreover, the D-LIOM can outperform Cartographer, which also supports various LiDARs, because of two reasons [102]. Firstly, it updates the IMU biases within its real-time optimization. Secondly, its loop detection method has a higher detection rate and initializes the relative poses based on feedback from multiple sources, which improves the establishment of the loop constraint.

- Hand-held mobile mapping

An open-source, open-hardware hand-held mobile mapping system has been proposed for large-scale surveys, featuring innovative software for extensive 3D mapping using an open-hardware hand-held measurement device [191]. This system is intended for educational and research purposes, aiming to make advanced mapping technology more accessible. The system utilizes a pose-graph loop closure technique and an ICP algorithm to minimize mapping errors. Initially, LiDAR odometry calculates the trajectory based on data from the mobile mapping device, which includes laser and IMU data. These data are synchronized to ensure that each 3D point has a corresponding orientation from the IMU. The next step is single-session refinement, where the trajectory is refined using pose-graph SLAM to reduce errors. Finally, multi-session refinement is conducted to further minimize errors between different mapping sessions, resulting in a consistent and accurate 3D map. The system produces 3D point clouds that can be applied in various real-world scenarios, such as city-level 3D mapping, cultural heritage preservation, creating ground truth data for mobile robots, precise forestry, and large-scale indoor 3D mapping.

## 5. Combination of LiDAR-Based and Vision-Based SLAM

**Figure 15:**
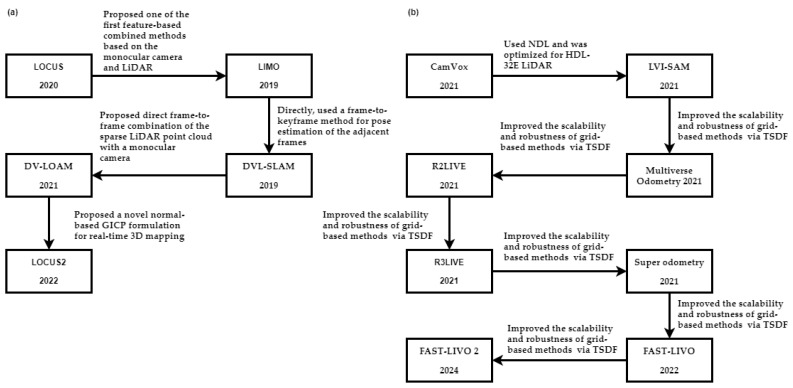
Combined SLAM methods’ evolution: (**a**) loosely and (**b**) tightly coupled.

Most LiDAR sensors provide a spare vertical distance measurement that may affect the loop detection process. As a result, LiDAR-based SLAM may fail in large and feature-poor environments with repeated patterns. On the other hand, vision-based methods have rich visual texture information. Consequently, the combination of the LiDAR-based and the vision-based methods improves the accuracy of the pose estimation by taking advantage of both methods [19]. This section describes combined methods and divides them into tightly and loosely coupled methods. The progress of the combined SLAM methods is illustrated in Figure 15.

### 5.1. Loosely Coupled Methods

This subsection describes two feature-based loosely coupled methods (LIMO [192] and LOCUS [193]) and two direct loosely coupled methods (DV-LOAM [194] and DVL-SLAM [195]). LOCUS is a direct method in the loosely coupled category.

- LOCUS

Sensor failure is a common issue in multi-sensor systems. To solve the problem, LiDAR odometry for consistent operation in uncertain settings (LOCUS) proposed a loosely coupled multi-LiDAR-IMU SLAM module with an integrated health-aware sensor in 2020 [193].

In the LOCUS, there are three main modules, namely, point cloud pre-processing, scan matching, and sensor integration. In its point cloud pre-processing step, the LOCUS corrects the raw LiDAR measurements’ motion distortion in motion distortion correction (MDC) units by using IMU data or odometry data such as the wheel, kinematic, and visual odometry, if available. After the correction step, point clouds are merged. The merged point cloud is then filtered to remove noise and out-of-range points, resulting in a lower computational load in the subsequent steps. LOCUS has two types of odometry sources, namely, primary and auxiliary. LiDAR odometry is the primary odometry source, which is used for pose estimation and initialized by auxiliary sources. Before initializing the pose estimation by other auxiliary sources in the sensor integration module, the health monitoring submodule evaluates auxiliary sensors’ data based on metrics such as feature counts, observability analysis, rate check >1 Hz, and covariance check. If any auxiliary sensor failure is detected by the health monitoring module, the system chooses the most accurate source based on a priority queue. There are two priority queues of auxiliary sensors for legged-based and wheel-based moving platforms, which are visual-inertial-odometry (VIO), kinematic-inertial-odometry (KIO), IMU, no input and VIO, wheel-inertial-odometry (WIO), IMU, no input, respectively. Remember that LiDAR is the primary sensor in both moving platforms. Finally, the scan matching module takes the pre-processed point cloud and sends it to an optional flat ground assumption (FGA) submodule. The FGA can either use IMU data to detect the flat ground or be enabled/disabled manually. The scan matching module uses GICP-based scan-to-scan and scan-to-submap methods. The GICP computes the optimal transformation matrix for scan-to-scan $\left(\hat{T}\right)$ and scan-to-submap $\left(\bar{T}\right)$ by:

(17) $$\hat{T_{k}^{k-1}}=argmin_{T_{k}^{k-1}}\epsilon \left(T_{k}^{k-1}L_{k},L_{k-1}\right)$$

(18) $$\bar{T_{k}^{k-1}}=argmin_{T_{k}^{k-1}}\epsilon \left(T_{k}^{k-1}L_{k},S_{k}\right)$$

where ϵ is the residual error, $L_{k}$ is the $k^{th}$ LiDAR scan, T is transforming matrix and $S_{k}$ is the local submap at time step k.

- LOCUS2

There is a second version of the LOCUS, called LOCUS2 [196]. LOCUS2, an advanced version of LOCUS, is a robust and computationally efficient LiDAR odometry system designed for real-time underground 3D mapping. While LOCUS is a multi-sensor LiDAR-centric solution for high-precision odometry and 3D mapping featuring a multi-stage scan matching module, LOCUS2 introduces several enhancements. These include a novel normal-based GICP formulation that reduces computation time for point cloud alignment, an adaptive voxel grid filter to maintain computational load regardless of the environment’s geometry, and a sliding window map approach to bound memory consumption. These improvements make LOCUS2 more suitable for deployment on heterogeneous robotic platforms involved in large-scale explorations under severe computation and memory constraints.

In LOCUS, loop closure is achieved through a multi-stage scan matching module that ensures high-precision odometry and 3D mapping. LOCUS2, however, enhances this process by incorporating the normal-based GICP formulation, which improves the robustness and accuracy of loop closure.

- LIMO

In 2019, LiDAR-monocular visual odometry (LIMO) proposed one of the first feature-based combined methods based on the monocular camera and LiDAR [192]. It starts with feature extraction and landmarking using the viso2 [197] library alongside deep learning. Then, it transforms the frame of LiDAR points into the camera frame and projects the points onto the image. Afterward, it uses a subset of points to detect local planes. The points surround an image feature point within certain borders. Considering features on the edges and corners in the image, it estimates the depth of features based on depth histograms with a fix bin of 0.3m. Next, it estimates pose changes relying on a frame-to-frame method using the perspective-n-point (PnP) problem [198]. To increase efficiency, it equally distributes near, middle, and far landmarks. In the same step, semantic information eliminates dynamic objects and regulates how important each landmark is. Finally, it selects keyframes when the mean optical flow is not larger than a certain value, which is followed by estimating transformations using keyframe-based bundle adjustment.

LIMO is fast and computationally efficient. However, it only uses LiDAR measurements to determine depths in the images and ignores other information in the LiDAR data. As a result, it suffers from accumulated errors in long-term runs [41] and is sensitive to noise [199], angle of view [200], and motion blur [201].

- DV-LOAM

Direct visual LiDAR odometry and mapping (DV-LOAM) proposed a combination of the sparse LiDAR point cloud with a monocular camera image in 2021 [194]. The DV-LOAM has a database and three main modules, namely, a two-staged visual odometry, a LiDAR mapping, and a parallel global and local search loop closure detection (PGLS-LCD).

To achieve better efficiency, the first module estimates the initial pose of the adjacent frames by taking the LiDAR points as salient feature points of the image in the same frames. Next, it optimizes the pose estimation by a sliding window-based method. The second module generates keyframes with the criteria of overlap ratio and time interval based on reference [202]. It also extracts geometrical features similar to the LeGO-LOAM. Then, geometrical features cancel the distortions of the point cloud and visual odometry drift errors to further refine the pose estimation. Finally, it performs scan-to-map optimization for every time stamp. The third module eliminates global drift by using both BoW and LiDAR-Iris [203] descriptors simultaneously to find loop closure scenarios. Next, it implements the TEASER algorithm for further correction of the pose estimation. As its last step, the PGLS-LCD refines the pose correction by V-GICP (see Section 3).

- DVL-SLAM

Direct visual-LiDAR SLAM (DVL-SLAM) (2019) [195] projects LiDAR points into images and tracks the images by minimizing the direct photometric error. The DVL-SLAM and DV-LOAM are remarkably similar. However, they have three differences. First, the DVL-SLAM uses a frame-to-keyframe method for pose estimation of the adjacent frames, but the DV-LOAM relies on a frame-to-frame method. Thus, the sliding window optimization refines the keyframe pose and each frame pose in the DVL-SLAM and DV-LOAM, respectively. Secondly, the DVL-SLAM takes LiDAR points limited by the field of the camera view whereas all points in the DV-LOAM. Lastly, the DVL-SLAM uses the BoW descriptor, while the DV-LOAM has the additional LiDAR-Iris within its PGLS-LCD module.

### 5.2. Tightly Coupled Methods

The tightly coupled SLAM methods in this subsection include features-based methods (CamVox, LVI-SAM, and R2LIVE), direct methods (R3LIVE and Fast-LIVO), and a hybrid method (Multiverse Odometry).

- CamVox

Camera-Livox (CamVox) (2021) integrates the Livox LiDAR depth measurements with an RGB camera to perform SLAM based on the ORB-SLAM 2 (RGB-D version) [204]. Compared to the ORB-SLAM 2, it uses LiDAR-assisted RGB-D images of each scene. It also has a separate RGB-D input pre-processing module and an uncontrolled automatic calibration module.

The CamVox’s pre-processing module first uses IMU data to correct the raw LiDAR distortions. Then, it generates a depth image by projecting LiDAR points to the RGB camera frame with respect to extrinsic calibration parameters. After that, it combines RGB and depth images to obtain an RGB-D image. When the moving platform is still, the uncontrolled automatic calibration module accumulates a few seconds of LiDAR scans. Next, it uses the initial extrinsic calibration to process the accumulated point cloud and RGB image. The process results in grayscale, reflectivity, and depth images. Then, it finds the corresponding edge features between the images based on the ICP method to perform further calibration.

In ORB-SLAM 2, key points are divided into two groups: close and far. Close points have an elevated level of depth certainty and are used to estimate scale, translation, and rotation. Far points, on the other hand, are only used for rotation estimation and are, therefore, less informative. Since LiDAR depth measurements are longer-ranged, denser, and more accurate compared to an RGB-D camera, the CamVox outperforms both ORB-SLAM 2 and VINS-Mono [204].

- LVI-SAM

In 2021, LiDAR-visual-inertial smoothing and mapping (LVI-SAM) combined LIO-SAM and VINS-Mono in a tightly coupled manner [19]. It initializes and corrects the odometry results of each one by the other one. If one module fails, LVI-SAM can still use the other one. For loop closure detection, the VIO module first detects the loop closures, and then the LIO module refines the detected loop closures. Consequently, it outperforms both VINS-Mono and LIO-SAM by a more accurate loop detection based on the developers’ results in outdoor environments with multiple loops and rich features [19]. This paper evaluates LVI-SAM’s performance in an EGD environment.

- Multiverse Odometry

Multiverse Odometry (2021) proposed a combined method that implements multiple odometry algorithms and then selects the superior odometry result [205]. The method consists of four modules, namely input pre-processing, odometry, sanity check, and scoring.

The pre-processing module takes data from a monocular camera and a 3D LiDAR. It extracts features of the monocular camera based on a SIFT descriptor [206] and uses the LiDAR point cloud in a voxelized form.

The next module takes the processed data and calculates transformations using multiple algorithms, including GICP, P2P-ICP, NDT, ColorICP, and Huang’s method [207]. The module also considers a constant velocity model to use last timestamp estimation without solely relying on sensors’ data.

The sanity check module then detects abnormal transform estimation by considering the dynamic and kinematic constraints of the moving platform. Moreover, it considers the Ackermann model [208] within kinematic constraints if the moving platform is a vehicle.

Finally, the scoring module calculates the Chamfer distance metric [209]. It uses an odometry algorithm’s results as a source for evaluating odometry results only if its transform estimation passes the sanity check. Based on the calculated Chamfer distances, it outputs the transformation that has the lowest score and stores it for initialization of the next iteration. Equation (19) gives the Chamfer distance formulation.

(19) $$D_{chamfer}\left(M,N\right)=1/M\sum_{m\in M}d_{N}\left(m\right)$$

where M and N are sets of points in the source and target point clouds, respectively. Given the transformation between M and N, d_N represents the minimum distance between point m and its nearest neighbor point in N. Since Multiverse Odometry implements multiple algorithms simultaneously, it outperforms each of them solely. It benefits accuracy and resiliency in terms of sensor failures [205].

- R2LIVE

Real-time, LiDAR-inertial-visual tightly coupled state estimator (R2LIVE) combined the Fast-LIO 1 and VINS-Mono in 2021 [210]. After receiving LiDAR scans, it extracts planar features, removes distortions, and calculates point-to-plane residuals. Simultaneously, it finds FAST corners, tracks them using Kanade–Lucas–Tomasi (KLT) [211] based on a visual landmarks map, and calculates re-projection errors for camera images. Next, it fuses the last step results of LiDAR and visual threads with IMU propagation in a tight manner based on an error-state iterated Kalman filter (ESIKF). Then, the ESIKF’s output updates the LiDAR features map and visual landmarks. For landmark refinement, it uses a factor pose graph optimization similar to the VINS-Mono but considers LiDAR pose estimations, too.

- R3LIVE

Robust, real-time, RGB-colored, LiDAR-inertial-visual tightly coupled state estimation (R3LIVE) was built upon R2LIVE in the same year [212]. R3LIVE uses Fast-LIO 2 and a different visual-inertial module. Many modules are adopted from R2LIVE and Fast-LIO 2. As part of its visual-inertia module, the input image is drawn with a certain number of global map points before state estimation. The estimator implements ESIKF and considers photometric errors based on the color of the map points, which does not vary with either camera translation or its rotation. Then, a frame-to-frame optical flow tracks map points before minimization of the PnP projection error. After that, it finds the minimum frame-to-map photometric error of map points. Finally, it utilizes estimated state and raw input to render textures and update the global color map. As a result, it does not need to extract salient points of input images, which makes it faster than R2LIVE. In addition, R3LIVE uses a faster LIO module (Fast LIO 2 instead of Fast LIO 1) that contributes to its faster performance compared to R2LIVE [210].

- FAST-LIVO

Fast and tightly coupled sparse-direct LiDAR-inertial-visual odometry (FAST-LIVO), which was introduced in 2022 [213], directly adopted Fast-LIO 2 as its LiDAR-based module and combined it with a vision-based module, which is like DVL-SLAM in that both project LiDAR points onto images and uses photometric error. However, DVL-SLAM only performs frame-to-frame image matching without the incorporation of IMU measurements or LiDAR registrations. In contrast, FAST-LIVO tightly couples sparse frame-to-map photometric error-based image matching with LiDAR registration point-to-plane residuals and IMU forward propagation using ESIKF state estimation. Moreover, it has an outlier rejection module aiming to improve LiDAR-to-image projection and overall system accuracy. The module removes a) points on the edges with disrupted depth information and b) blocked points in the image view. It outperforms R2LIVE, Fast-LIO 2, and DVL-SLAM according to the developers’ findings using the NTU VIRAL dataset [214].

- FAST-LIVO 2

FAST-LIVO2 was developed as a fast, direct LiDAR-inertial-visual odometry framework designed to achieve accurate and robust state estimation in SLAM tasks, making it suitable for real-time, onboard robotic applications [215]. It fuses IMU, LiDAR, and image measurements through an ESIKF, addressing the dimension mismatch between heterogeneous measurements with a sequential update strategy in the Kalman filter. The framework employs direct methods for both visual and LiDAR fusion, utilizing a unified voxel map for integrating geometric structures and image patches. Enhancements in image alignment accuracy and robustness are achieved through plane priors from LiDAR points and real-time image exposure estimation.

- Super Odometry

Super odometry is an advanced estimator that uses IMU-centric LiDAR-visual-inertial technology [216]. It is known for its robust performance in difficult settings. The system’s architecture is centered around incorporating an IMU along with LiDAR and visual data. This integration guarantees accurate localization and mapping, even in situations when the visual data are damaged, or the performance of the LiDAR is impaired. The fusion technique improves the system’s ability to withstand various situations, such as low-light or dynamic surroundings, which are typically challenging for standard SLAM systems. Super Odometry demonstrates its versatility and reliability in the dynamic field of autonomous navigation and robotics, especially in challenging or unpredictable environments, by giving priority to IMU data. This ensures consistent and accurate estimations of posture and trajectory. Super Odometry combines the pros of loosely coupled and tightly coupled methods and recovers motion in a coarse-to-fine manner.

## 6. NeRF-Based SLAM

Neural radiance fields (NeRF)-based SLAM is a cutting-edge approach that merges the capabilities of NeRF and SLAM to improve mapping and localization tasks. NeRF employs deep learning to depict a scene as a continuous 3D function, enabling the generation of new views from various angles [217]. SLAM, conversely, is a method used in robotics and computer vision to construct a map of an unfamiliar environment while concurrently tracking the device’s position within that space. By combining NeRF with SLAM, researchers aim to achieve more precise and detailed 3D reconstructions and real-time localization.

The operation of NeRF-based SLAM consists of two primary components: tracking and mapping. During the tracking phase, the system refines the camera pose and the parameters of the implicit scene representation using inverse rendering techniques. This involves calculating the photometric loss between the rendered image and the actual image captured by the camera and then adjusting the camera pose and scene parameters to minimize this loss. In the mapping phase, NeRF-based SLAM utilizes neural implicit representations and volume rendering functions to generate a detailed 3D map of the environment. This map can be continuously updated and refined as new data are gathered, allowing for real-time adjustments and enhancements [218].

One of the key benefits of NeRF-based SLAM is its ability to produce high-fidelity 3D maps with rich geometric and photometric details. This is particularly advantageous in applications such as autonomous driving, robotics, and augmented reality, where accurate and detailed environmental information is essential. Additionally, NeRF-based SLAM can handle complex scenes with varying lighting conditions and textures, which traditional SLAM methods often struggle with [219].

NeRF-based SLAM faces several challenges, including high computational requirements for training and rendering, which hinder real-time performance [220], generalization and scalability [221], and integrating NeRF with existing SLAM methods [221].

## 7. Benchmarking

The comparison of SLAM methods is a crucial aspect of evaluating their performance across diverse environments, particularly under challenging conditions. Most scenarios in robotic-based nondestructive evaluation of infrastructures involve enclosed and GPS-denied (EGD) conditions. To assess the effectiveness of the reviewed methods in EGD conditions, a quantitative analysis was conducted. We used two datasets: (1) our collected dataset and (2) a publicly available dataset collected in Luleå, Sweden [222]. Table 2 and Table 3 provide a list of the evaluated SLAM methods, categorizing them by approach and presenting their performance results on the selected test dataset. Only robot operating system (ROS)-based methods were considered for benchmarking. The following subsections describe the dataset utilized for evaluation and discuss the results achieved by the various methods.

### 7.1. Luleå Dataset

#### 7.1.1. Dataset Details

**Figure 16:**
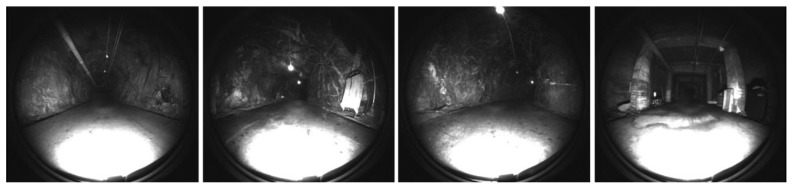
Distinct locations of EGD Conditions in the Luleå Tunnel Dataset: The images demonstrate the poorly illuminated and feature-poor characteristics of the underground tunnel environment, highlighting the challenging conditions for SLAM algorithms.

The study evaluates vision-based, LiDAR-based, and combined SLAM methods using a dataset collected in an underground tunnel in Luleå, Sweden [222], the only LiDAR-visual-inertial available open-source dataset exhibiting EGD characteristics at the time of this research. The dataset was collected in a tunnel environment characterized by extremely low-light conditions, feature-poor surfaces, and complex geometric layouts, providing a rigorous testbed for SLAM systems. These conditions include poorly illuminated sections with minimal light sources, resulting in areas that are nearly pitch dark, and regions where the tunnel walls are uniform, offering limited distinguishable features necessary for traditional SLAM approaches. The dataset satisfies EGD conditions by presenting environments that are poorly illuminated and feature-poor, as shown in Figure 16. The scenes exhibit minimal light sources, creating areas with low visibility, and the uniform tunnel walls offer sparse, distinguishable features. These conditions pose significant challenges for SLAM methods, particularly in accurately tracking and mapping in GPS-denied, low-texture environments.

The LiDAR-visual-inertial dataset comprises point clouds captured using a 3D LiDAR (Velodyne Puck Lite) and two 2D LiDARs (RPLiDAR S1 and RPLiDAR A2). Vision data were collected using multiple sensors, including an RGB camera (Intel RealSense T265), an RGB-D camera (RealSense D455), an event-based camera, and a 360-degree camera (Prophesse EVALUATION KIT Gen3M VGA-CD 1.1), providing a comprehensive set of visual inputs in various light conditions. Additionally, inertial data were gathered using a VectorNav IMU, enhancing the robustness of motion estimation in challenging scenarios. The dataset is further augmented with three distinct sensor setups, combining infrared, vision, IMU, and UWB ranging sensors, divided into three sections labeled AB, BC, and CD, each representing distinct environmental conditions within the tunnel.

The tunnel’s lack of natural features and sparse textures significantly challenge feature extraction and matching processes in SLAM, while the poor illumination and corridor bending pose difficulties for visual-based methods. These challenging conditions make this dataset highly suitable for benchmarking SLAM algorithms, as it accurately reflects real-world EGD scenarios where many SLAM solutions struggle.

**Figure 17:**
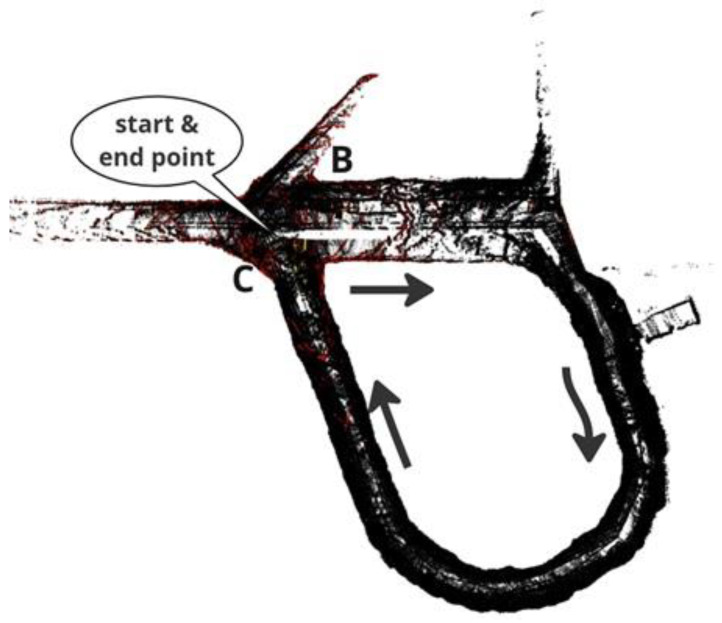
A top view of the map generated from the 3D LiDAR scans from the field test environment.

Figure 17 depicts the top-down view of the path used to evaluate the reviewed methods. The path for evaluating SLAM methods begins at point B and ends at point C. The path includes a curve that returns to the start point, abrupt rotations, EGD conditions, and dust in some places. The recording dataset is 181 s (about 6 min) traversing inside the path from B to C. The arrows show directions.

**Figure 18:**
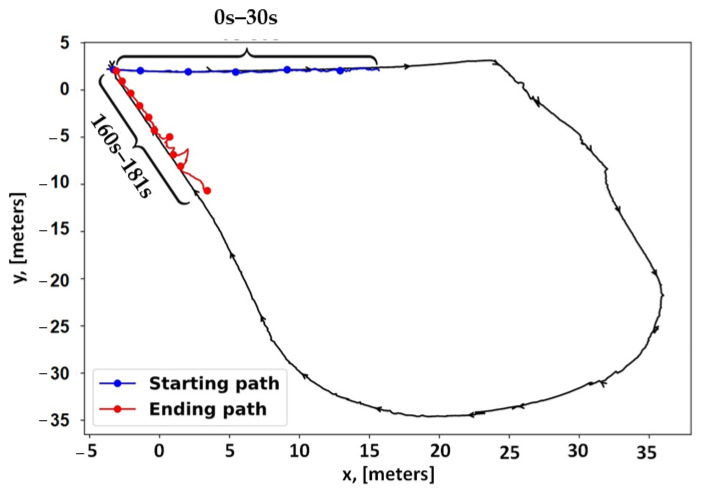
Traversing path and available ground truth data.

The ground truth is recorded by the MDEK1001 ultra-wideband sensor that provides a positioning accuracy of 10 cm (about 3.94 in), and the anchor of sensors provides an accuracy of a few centimeters [223]. The ground truth is available between seconds 0–30 and 160–181, see Figure 18. Since there are three sensor setups in the provided dataset, sensor setup number 2 works for LiDAR-based methods and VINS-Mono, which supports a mask for the fisheye camera. However, for the vision-based methods, sensor setup number 3 works better since the RealSense D455 is included in the setup. The LiDAR-based methods and VINS-Mono are compared with the ground truth provided, and the vision-based methods are compared with the LVI-SAM, a LiDAR-based method that owns the least RMSE.

#### 7.1.2. Results

**Figure 19:**
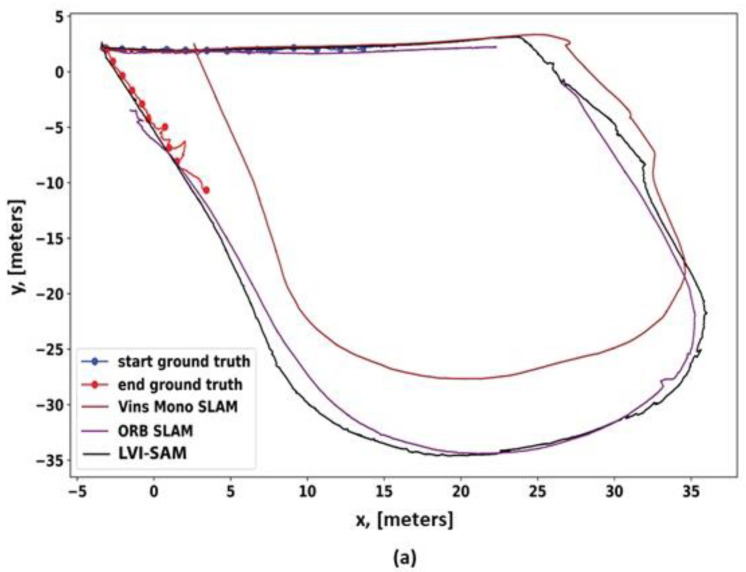

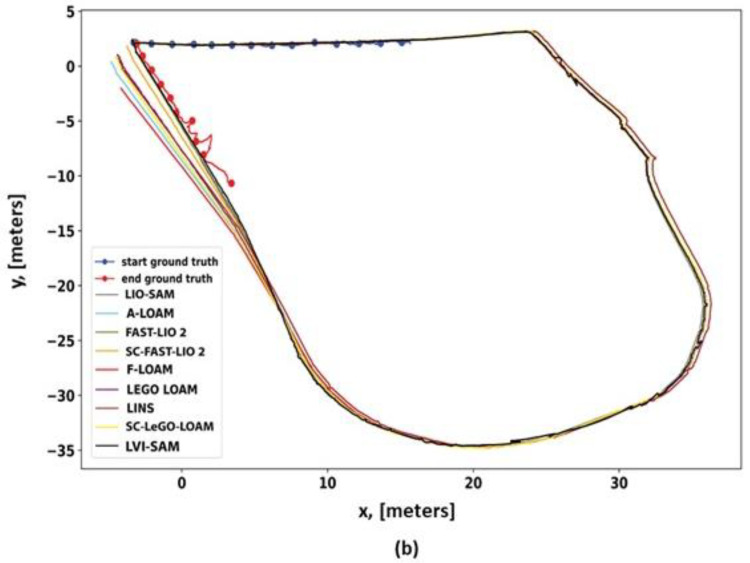
Trajectory results for the evaluated methods. (**a**) The trajectory of vision-based methods compared to ground truth and LVI-SAM. (**b**) The trajectory of LiDAR-based methods compared to ground truth.

This paper presents the results of evaluations in terms of RMSE for trajectory error, translation error, and resource consumption for memory and CPU. The trajectory obtained by various methods is compared with the ground truth in Figure 19. Figure 19a depicts the results for vision-based methods, and Figure 19b displays the trajectory results obtained by LiDAR-based approaches. The line with a circle marker indicates the ground truth data, and each method is depicted in a specific color and corresponding legend.

Since a sequence of images cannot be used to estimate real-world depth in monocular SLAM, the results for ORB-SLAM need to be scaled to fit into the real-world trajectory. Additionally, the vehicle contains an extreme rotation at the end of the straight path turning into a curved tunnel. The path in the dataset is wider at the start and enters a tight tunnel after 45 s until the end of the dataset. This results in the loss of feature tracking in ORB-SLAM. Since ORB-SLAM demonstrates an acceptable performance in the curved tunnel, we have evaluated the method in straight and curved paths separately and combined the results together for evaluation. Consequently, the results for ORB-SLAM include an inconsistency at the top right corner in Figure 19a. The path for evaluating ORB-SLAM is the same as other methods with the turning corridor removed. It should be considered that although ORB-SLAM demonstrates better performance than VINS-Mono, the VINS-Mono passes the corridor without error. The evaluation path for ORB-SLAM starts in the mentioned sections separately and is compared to LVI-SAM which owns the least RMSE compared to other methods in LiDAR-based methods.

- Trajectory root mean square error

**Figure 20:**
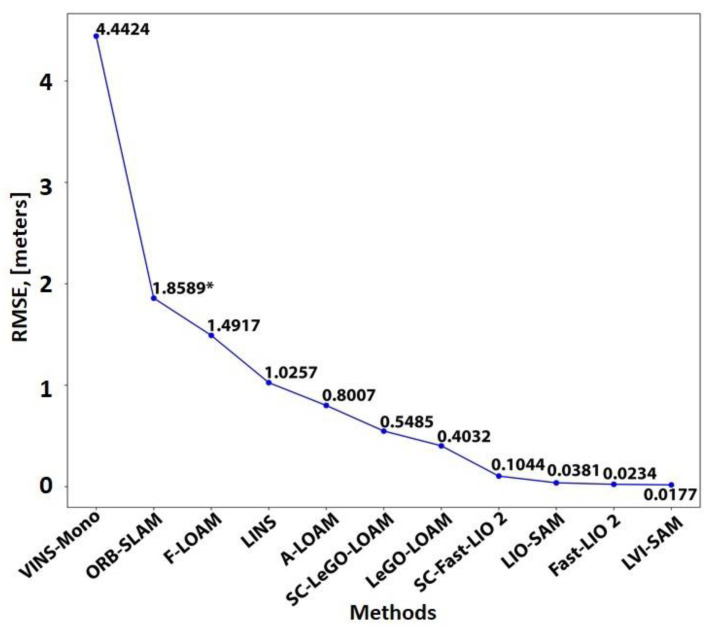
Trajectory RMSE of evaluated methods for 181 s (about 6 min) of traversing the mine environment. (* considering that ORB-SLAM fails to turn in the corridor that contains quick rotation resulting in failure of feature tracking).

This study uses RMSE as an index for quantitative analysis of trajectory error for the evaluated methods. Figure 20 compares the RMSE results for both LiDAR-based and vision-based methods from trajectories of Figure 18 and Figure 19. According to the conducted evaluations, ORB-SLAM demonstrated superior performance to VINS-Mono. However, the ORB-SLAM failed to compensate for sudden rotations without an IMU. As a result, it caused blurred images, which resulted in failure in tracking.

As discussed in Section 5, the LVI-SAM combines LIO-SAM and VINS-Mono to improve the overall performance. Therefore, it achieved the best result with only 1.8 cm (about 0.71 in) RMSE compared to 3.8 cm (about 1.5 in) and 442.2 cm (about 14.51 ft) of the LIO-SAM and VINS-Mono, respectively.

The Fast-LIO 2 had the second-best RMSE of 2.3 cm (about 0.91 in), which is almost 45 times better than the LINS. A massive gap between the performance of the two methods was expected because Fast-LIO 2 is not limited to feature extraction and has a simpler KF (based on Fast-LIO 1), as mentioned in Section 4.2. Surprisingly, the LINS was also outperformed by A-LOAM, LeGO-LOAM, and SC-LeGO-LOAM which do not use IMU measurements. This could be because the LINS is sensitive to the IMU state at the start point and relies on a sparse point cloud for pose estimation and mapping.

Among the LiDAR-only methods, the LeGO-LOAM had two times less RMSE than the A-LOAM due to its loop detection module. However, the A-LOAM outperformed the F-LOAM by a 69.1 cm (about 2.27 ft) difference in RMSE since the F-LOAM considers a linear estimation within its system. Another unexpected result in Figure 20 is that the SC integration deteriorates RMSE. One explanation can be that the used dataset includes a few view changes.

- Translation error

**Figure 21:**
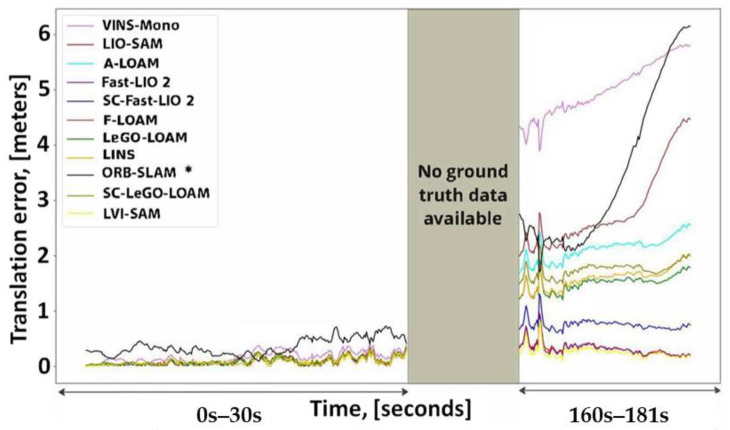
Translation error for the evaluated methods against ground truth (* considering that ORB-SLAM failed to follow the turn in the corridor containing a quick rotation. The failure was due to feature tracking failure caused by blurry image).

The translation error is computed using the available ground during 0–30 s of the starting path and 160–181 s (about 6 min) of the path ending. Figure 21 compares the computed translation errors. As expected, the results follow a remarkably similar pattern to Figure 19.

- Resource consumption

**Figure 22:**
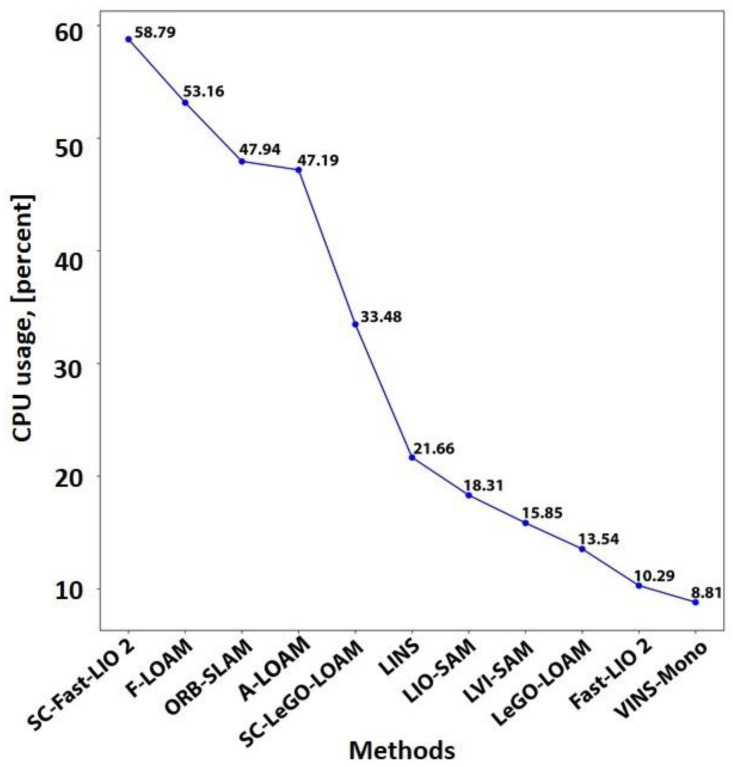
Mean CPU usage consumption for 181 s (about 6 min) traversal time.

**Figure 23:**
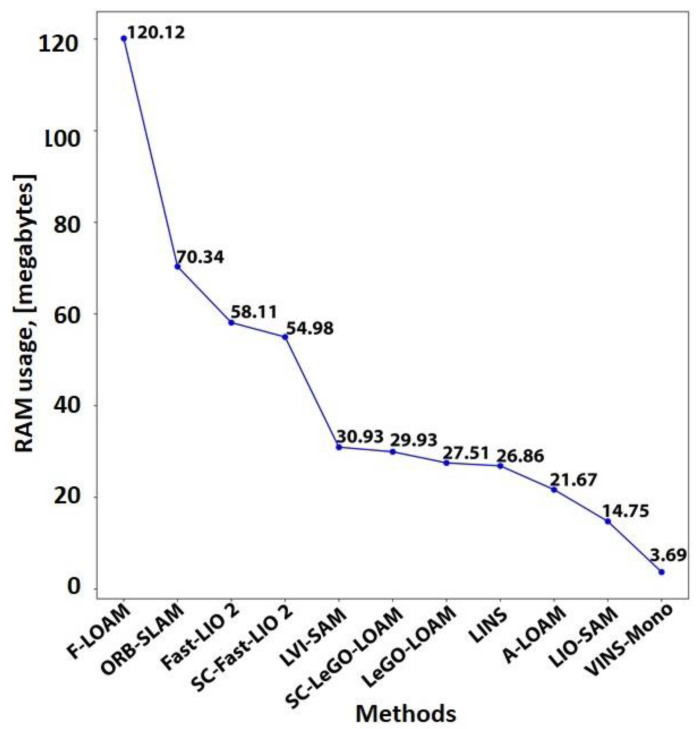
Mean memory usage for 181 s (about 6 min) traversal time.

To analyze the resource consumption of the evaluated methods, this study tracks the summation usage of all threads belonging to the SLAM method from both the central processing unit (CPU) and random-access memory (RAM) using a publicly available repository [224]. Our hardware specifications were as follows: Intel^®^ Core™ i3-3120M CPU, 16 gigabytes of DDR3 RAM, and 500 gigabytes SSD hard drive. CPU and RAM usage are recorded for 181 s (about 6 min) of traversing, and the mean consumption value for each resource is calculated. Figure 22 and Figure 23 show CPU and RAM usage, respectively. The fastest LiDAR-based method is the Fast-LIO 2, as it uses a simpler KF than the LINS and relies on the iK-D tree method. Thus, it requires less computational costs. The LeGO-LOAM is the second fastest LiDAR-based method. Although the LeGO-LOAM added a loop detection module to the LOAM, it is 3.3 times faster than the A-LOAM. The main reason is that the LeGO-LOAM uses K-D tree data structure and iSAM2 optimization to be more computationally efficient. While the F-LOAM aimed to be faster, it is nearly 6 percent slower than the A-LOAM. Among the LiDAR-inertial methods, the LIO-SAM is slightly faster than the LINS with only a 2.46 percent difference because both methods perform scan matching locally. The LINS and LIO-SAM are based on the LeGO-LOAM and include extra modules compared to it. Thus, both methods are slower than the LeGO-LOAM. Similarly, the SC integration has made the base method slower. It slowed down the Fast-LIO 2 and the LeGO-LOAM by 5.7 and 2.5 times, respectively. In the vision-based methods, the VINS-Mono is 5.4 times faster than the ORB-SLAM due to sparse feature points and point clouds compared to ORB-SLAM. It is also the fastest method in total. Finally, the combination of the VINS-Mono and LIO-SAM, the LVI-SAM, is placed between the two methods regarding CPU usage. The LVI-SAM has more modules compared to the LIO-SAM. However, it is faster due to the better initialization step.

Figure 23 shows that all methods required less than 121 megabytes of RAM without considering the operating system (OS) and other running programs. Most methods used less than 31 megabytes of RAM, while alleged ‘Fast’ methods used more RAM. Unlike CPU usage, SC integration slightly affected RAM usage. Similar to Figure 22, the VINS-Mono achieved the best result with only 3.69 megabytes of RAM usage. However, the LVI-SAM uses more RAM than the LIO-SAM and VINS-Mono because it needs to maintain both methods’ data in RAM while processing them. Since even tiny computers are equipped with at least 4 gigabytes of RAM nowadays, RAM used by the SLAM methods would not be a concern compared to the OS and other running programs in short-term runs.

### 7.2. Our Dataset

#### 7.2.1. Dataset Details

**Figure 24:**
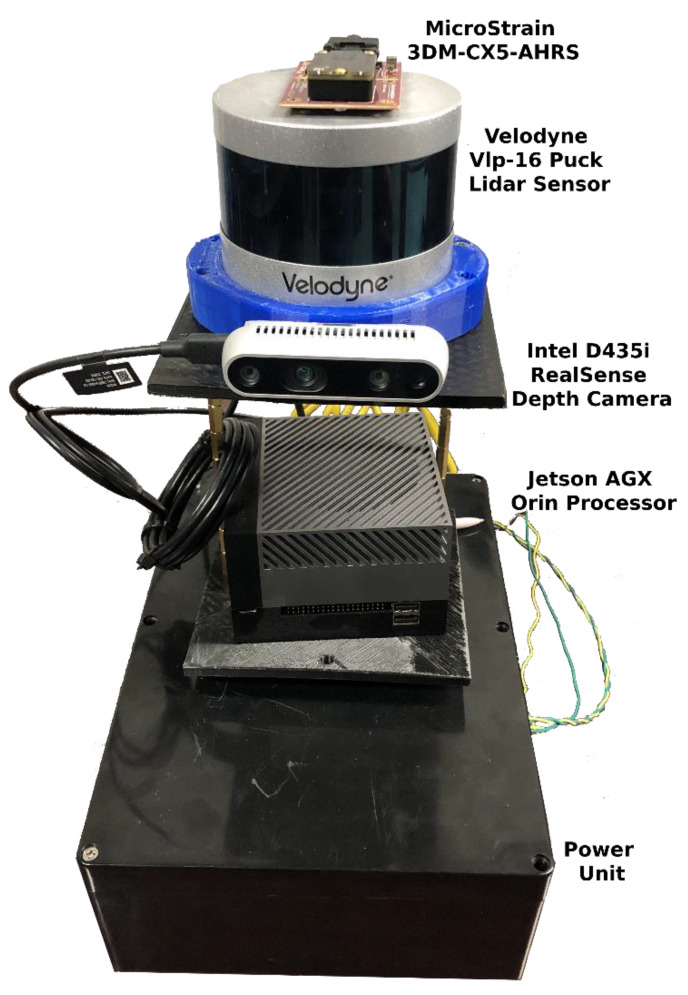
Data collection device.

The handheld dataset was collected in the campus basement of Drexel University, which presents characteristics of poor lighting and a textureless environment. The traversed path is a large loop with a length of approximately 50 m. Data collection was conducted using a setup comprising a depth camera, a LiDAR sensor, and an IMU, as illustrated in Figure 24. Table 4 summarizes each sensor’s information.

#### 7.2.2. Results

**Figure 25:**
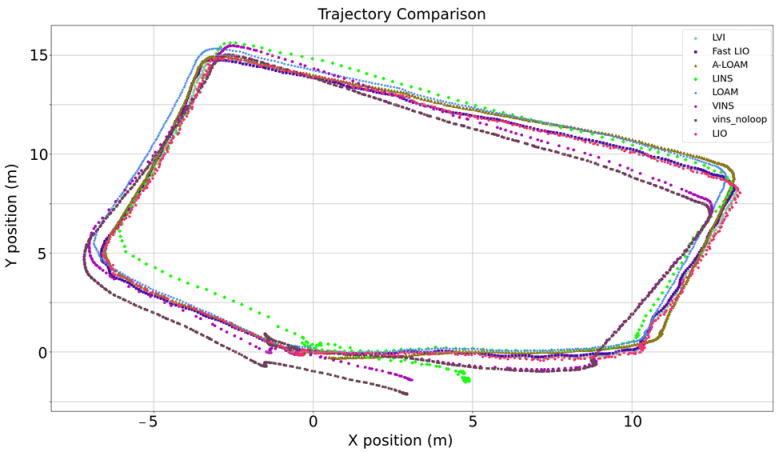
Traversed path using data collection device to SLAM methods.

**Figure 26:**
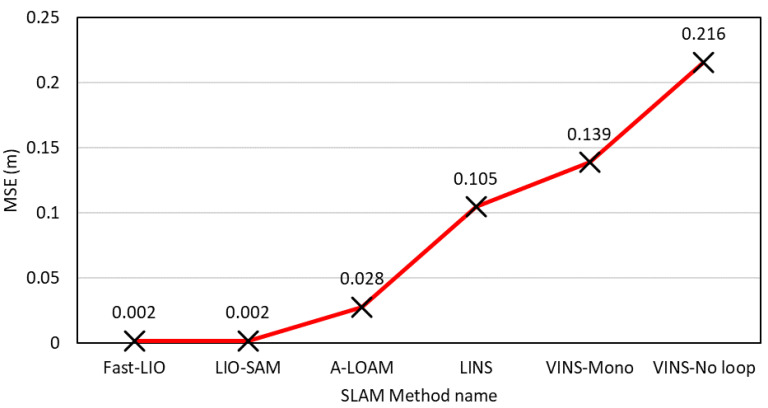
MSE values for different SLAM methods compared to LVI SAM as ground truth.

Various SLAM methods were evaluated, from visual, visual-inertial, LiDAR, LiDAR-inertial, and LiDAR-visual-inertial methods. Visual SLAM was observed to fail in tracking motion during corridor turns, particularly under texture less conditions, due to inadequate detection of features. The performances of LINS, VINS-Mono, VINS-No loop, LIO-SAM, LVI-SAM, Fast-LIO, and A-LOAM were assessed using the collected dataset. Among the evaluated methods, LVI-SAM again exhibited the lowest RMSE using our dataset. Consequently, LVI-SAM was selected as the ground truth for odometry comparisons. The traversal paths of the evaluated methods are illustrated in Figure 25. Moreover, the mean square error (MSE) values, comparing each method against the ground truth, are presented in Figure 26.

**Figure 27:**
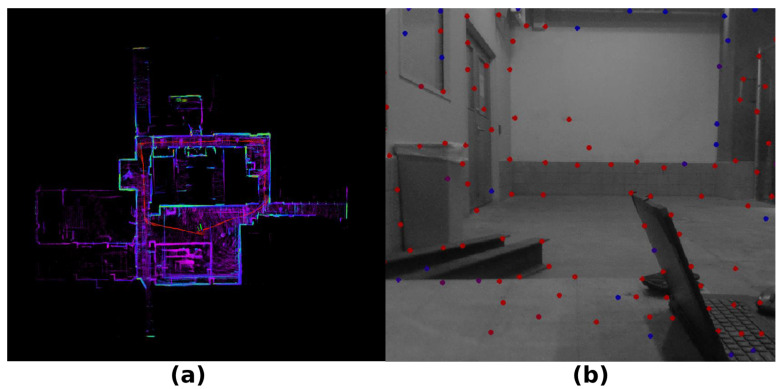
(**a**) LiDAR-based mapping and odometry results. (**b**) Feature tracking is performed using the VINS-Mono SLAM method. Red dots indicate newly detected features in the current frame, blue dots represent successfully tracked features across frames, and purple dots denote features that failed to track or were rejected as outliers.

Figure 27 presents the results of mapping and odometry of LiDAR-inertial and VI methods. Figure 27a LiDAR-based mapping and odometry, showcasing environmental mapping and localization, and Figure 27b visual-inertial (VI) SLAM feature tracking where red dots represent newly initialized features in the current frame, blue dots signify successfully tracked features across consecutive frames, and purple dots correspond to features that failed to track or were excluded as outliers in a poor lighting environment.

## 8. Conclusions

From an infrastructure inspection perspective, this study meticulously examined vision-based, LiDAR-based, and combined SLAM methods, evaluating their performance using our dataset and a test dataset characterized by enclosed and GPS-denied conditions. Our findings indicate that LiDAR-based methods excelled in these challenging conditions, and two vision-based methods (ORB-SLAM2 and VINS-Mono) also achieved acceptable performance, contrary to common presumptions. The performance of LiDAR-based SLAM methods was superior to vision-based SLAM, albeit at the cost of higher computational power and expense. The integration of VINS-Mono with LIO-SAM in the LVI-SAM algorithm resulted in the lowest RMSE and translation errors, highlighting its robustness. This study reviewed open-source methods that had at least one peer-reviewed or technical publication, except for methods that failed to meet the criteria but were necessary to explain qualified methods. Additionally, only ROS-based methods were included in the benchmarking process. This study provides valuable insights into the application of SLAM for infrastructure inspection, particularly in enclosed and GPS-denied environments. However, there are a few limitations that need to be highlighted for future improvement and research. Our datasets aimed to simulate an enclosed and GPS-denied environment for infrastructure inspection with a limited number of sensors. Consequently, future research should focus on testing additional datasets (e.g., SubT datasets) or collecting new datasets with diverse types of sensors (e.g., solid state LiDARs) to further investigate SLAM at infrastructure inspection service. Furthermore, we encountered challenges with some packages that were damaged or whose GitHub repositories were no longer available at the time of this study. Despite our best efforts, we were unable to run some methods. The current paper benchmarked SLAM under the EGD condition presented in most infrastructure evaluation scenarios. A future work might investigate SLAM performance concerning various parameters, such as different point cloud densities and measurement techniques (i.e., terrestrial and mobile laser scanning). Furthermore, a future extension can identify and address specific issues of existing works within specific conditions (e.g., EGD) and consider exploring the possibility of requesting code or data directly from authors that do not have open-source repositories to broaden our coverage.

## Figures and Tables

**Table 1:** Previous relevant surveys in chronological order and the focus of study.

Publication Year	Focus	Vision-Based	LiDAR-Based	GPS-Denied
2008	Filtering methods and map consistency in large environments [21]	✓	x	x
2011	Visual odometry with monocular and stereo cameras [22]	✓	x	x
2012	Feature detection and feature tracking algorithms for pose estimation [23]	✓	x	x
2015	SLAM components and RGB-D methods [24]	✓	x	x
2015	SLAM components and image matching for loop closure detection [25]	✓	x	x
2015	Holistic review of mapping in vision-based SLAM [26]	✓	x	x
2016	Robustness, scalability, representation, and trends in SLAM [27]	✓	x	x
2017	Key frame-based SLAM with monocular camera [28]	✓	x	x
2017	Feature-based, direct methods, and RGB-D methods in SLAM [29]	✓	x	x
2018	SLAM in dynamic environments, structure from motion, and visual SLAM [30]	✓	x	x
2018	Direct and indirect methods, visual SLAM in dynamic environments [31]	✓	x	x
2018	Visual-inertial SLAM, filtering and optimization methods [32]	✓	x	x
2019	SLAM elements and semantic SLAM [33]	✓	x	x
2020	Deep reinforcement learning in visual SLAM [34]	✓	x	x
2020	SLAM dividing feature-based SLAM into high, middle, and low-level divisions [18]	✓	x	x
2020	Visual-LiDAR fusion-based SLAM [16]	✓	✓	x
2021	Precise radio frequency-based localization [35]	x	x	x
2021	Visual and visual-inertial SLAM and evaluation for VINS-Mono, ROVIO, ORB-SLAM 2, DSO, and LSD-SLAM [36]	✓	x	x
2021	Loop closure detection and deep learning-based loop detection [37]	✓	✓	x
2022	Learning-based semantic scene understanding [38]	✓	x	x
2022	SLAM systems used in a subterranean (SubT) challenge [39]	x	✓	✓
2022	LiDAR-based multi-sensor fusion SLAM works and evaluation of A-LOAM, LeGO-LOAM, SC-LeGO-LOAM, LIO-SAM, and F-LOAM [40]	x	✓	x
2022	Single and multi-sensor SLAM methods [41]	✓	✓	x
2022	Mono and multi-modal SLAM methods without benchmarking [42]	✓	✓	✓
2023	Visual SLAM integration with semantic segmentation and deep learning [43]	✓	x	x
2024	Visual SLAM in dynamic environment [44]	✓	x	x
2024	SLAM in extreme environments without benchmarking [39]	✓	✓	✓

✓ means yes, and x means no.

**Table 2:** List of evaluated vision-based methods and corresponding results.

Algorithm	Methodology	Result	Application
* Mono-SLAM	VFB	Failed in feature detection and tracking	First vision-based method
* PTAM	VFB	Failed in initialization for ground floor	Tracking and mapping optimization by multi-threading
+* S-PTAM	VFB	Authors could not run the code	Map and pose graph optimization by loop detection and bundle adjustment
+* OV2SLAM	VFB	Authors could not run the code	Mapping optimization with no need for offline training biases
* ORB-SLAM ^1^	VFB	Success	Tracking optimization using resistant-to-noise features
+* DTAM	VD	No publicly available repository	First dense tracking and mapping
* LSD-SLAM	VD	Failed in feature tracking	Computational optimization
* SVO	VD	Failed in tracking	Computational and performance optimization
+* DSO	VD	Authors could not run the code	Performance optimization
+* Kinetic Fusion	VRGB-D	No publicly available repository	First RGB-D method
* Dense visual SLAM	VRGB-D	Failed in feature detection and tracking	Localization optimization using keyframe selection and loop closure detection
+* Elastic Fusion SLAM	VRGB-D	Inconsistent repository	Mapping and global consistency optimization
+* Realtime onboard VI estimation	VIL	No publicly available repository	First real-time on-board visual-inertial method
+* Multi-sensor fusion	VIL	No publicly available repository	Sensor fusion optimization
+* SOFT-SLAM	VIL	No publicly available repository	Computational optimization by decreasing the IMU intervals
* MSCKF	VIT	Failed in tracking	First VI method
+* ROVIO	VIT	Authors could not run the code	Localization optimization
* OKVIS	VIT	Failed in tracking	Localization optimization using robust keyframe matching
+* VIORB	VIT	Inconsistent repository	Localization optimization by adding loop closure detection
+* S-MSCKF	VIT	No publicly available repository	Localization optimization by adding loop closure
* VINS-Mono	VIT	Success	Localization and pose graph optimization for global consistency
+* STCM-SLAM	VIT	No publicly available repository	Localization optimization in indoor environment
* Kimera	VIT	Failed due to feature detection and tracking	First VI method that added 3D mesh reconstruction
+* Yolo-SLAM	DVIT	SLAM for dynamic environments	Performance optimization in dynamic environments

^1^ Authors could not run ORB-SLAM 3. Thus, the original one was used. * Incompatible with VLP-16. + Not integrated with ROS. VFB: Visual feature-based, VD: Visual direct, VRGB-D: Visual RGB-D, VIL: Visual-inertial SLAM loosely coupled, VIT: Visual-inertial SLAM tightly coupled, DVIT: Deep learning-based and visual-inertial SLAM tightly coupled.

**Table 3:** List of evaluated LiDAR-based and combined methods and corresponding results.

Algorithm	Methodology	Result	Application
LOAM and A-LOAM	LF	Success	Cost-effectiveness
* LOAM-Livox	LF	Incompatible with VLP-16	Solid-state LiDAR
F-LOAM	LF	Success	Computational effectiveness
LeGO-LOAM	LF	Success	Ground vehicles
ISC-LOAM	LF	Failed in trajectory estimation	ISC integration
SC-LeGO-LOAM	LF	Success	SC integration
* Optimized-SC-F-LOAM	LF	Incompatible with the testing dataset	SC integration
+ PIN-SLAM	LF	Not integrated with ROS	Global map consistency
* M-LOAM	MLF	Incompatible with VLP-16	Implementing multi LiDARs
MULLS	ALF	Failed in trajectory estimation	Adapting to various LiDARs
LOL	DLF	Failed in trajectory estimation	Vehicles in urban environments
* SegMap	DLF	Authors could not run the code	Extracting segments of 3D point clouds
* SuMa++	DLD	Authors could not run the code	Integrating semantic information
* SuMa	LD	Authors could not run the code	Using surfels for resource efficiency
+* IMLS-SLAM	LD	No publicly available repository	Low-drift SLAM that relies solely on LiDAR data
* HDL-Graph-SLAM	LD	Incompatible with VLP-16	HDL LiDARs
* LIOM	LITF	Inconsistent repository	Long-term usage
LIO-SAM	LITF	Success	Real-time factor graph SLAM
LINS	LITF	Success	ROS-compatible light robots
* LiLi-OM	LITF	Incompatible with VLP-16	Both solid-state and conventional LiDARs
Fast-LIO 1	LITF	The repository is no longer available	Computationally efficiency and robustness
Fast-LIO 2 and SC-Fast-LIO 2	ALITD	Success	Adaptively using raw LiDAR data
* D-LIOM	AMLITD	Inconsistent repository	Adaptively using raw multi-LiDAR data
+ Hand-held mobile mapping	LITF	Not integrated with ROS	Versatile hand-held usage
* HectorGrapher	LILF	Inconsistent repository	Continues time SLAM for mobile rescue robots
* Cartographer	LILF	Authors could not run the code	2D and 3D SLAM across multiple platforms
* LOCUS and LOCUS 2	MLILD	Authors could not run the code	Subterranean and challenging situations
* CamVox	LVITF	Incompatible with VLP-16	Cost-effectiveness and solid-state LiDAR compatibility
LVI-SAM	LVITF	Success	Real-time factor graph SLAM
* R2LIVE	LVITF	Incompatible with VLP-16	Robust and real-time multi-sensor fusion SLAM
* R3LIVE	LVITD	Incompatible with VLP-16	Built upon R2LIVE with RGB-colored mapping
* FAST-LIVO(s)	LVITD	Incompatible with VLP-16	Real-time multi-sensor SLAM for onboard usage
* LIMO	LVLF	Authors could not run the code	Pioneer feature-based multi-sensor SLAM for autonomous driving
* DV-LOAM	LVLD	Inconsistent repository	Multi-sensor SLAM with PGLS-LCD for autonomous driving
* DVL-SLAM	LVLD	Inconsistent repository	Multi-sensor SLAM for sparse depth data with small FoV
* Multiverse Odometry	LVM	Inconsistent repository	Multi-odometry for autonomous driving
+* Super Odometry	LVIIC	No publicly available repository	IMU-centric multi-sensor SLAM for challenging situations

* Incompatible with VLP-16. + Not integrated with ROS. LF: LiDAR feature-based, MLF: Multi LiDAR feature-based, ALF: Adaptive LiDAR and feature-based, DLF: Deep learning and LiDAR feature-based, DLD: Deep learning-based and LiDAR direct, LD: LiDAR direct, LITF: LiDAR-inertial; tightly coupled and feature-based, ALITD: Adaptive LiDAR-inertial; tightly coupled and direct, LILF: LiDAR-inertial; loosely coupled and feature-based, MLILD: Multi LiDAR-inertial; loosely coupled and direct, LVITF: LiDAR-vision-inertial; tightly coupled and feature-based, LVITD: LiDAR-vision-inertial; tightly coupled and direct, LVLF: LiDAR-vision; loosely coupled and feature-based, LVLD: LiDAR-Vision; loosely coupled and direct, LVM: LiDAR-vision with multiple odometry methods, LVIIC: LiDAR-Visual-Inertial; IMU-centric. AMLITD: Adaptive multi-LiDAR-inertial; tightly coupled and direct.

**Table 4:** Details of the data collection setup’s sensors.

Sensory Data	Sensor	Sensor Information
RGB-D	Intel RealSense D435i (Intel, Santa Clara, CA, USA)	Resolution: 1920 × 1080 (RGB), 1280 × 720 (IR) Frame Rate: Up to 90 fps Depth Field of View: 85.2° × 58° Depth Range: 0.105 m to 10 m+ Accuracy: ±2% at 2 m distance
3D scan	Velodyne Puck VLP16 (Velodyne, San Jose, CA, USA)	Channels: 16 Range: Up to 100m (about 328.08 ft) Accuracy: ±3 cm (about 1.18 in) Field of View: 360° horizontal, 30° vertical Angular Resolution: 0.1–0.4° horizontal, 2.0° vertical Rotation Rate: 5 Hz–20 Hz Points per Second: ~300,000 (single return), ~600,000 (dual return)
Acceleration	MicroStrain 3DM-CX5-AHRS (MicroStrain, Williston, VT, USA)	Sampling Rate: Up to 1000 Hz (IMU), up to 500 Hz (EKF) Accuracy: ±0.25° RMS roll and pitch, ±0.8° RMS heading Resolution: <0.01° Operating Temperature: −40 °C to +85 °C

## Data Availability

Data is available on GitHub (https://github.com/arvinebr/SLAM_review).

## Nomenclature

**Abbreviation**		
2D	2 dimensional	
3D	3 dimensional	
4D	4 dimensional	
6D	6 dimensional	
AI	Artificial intelligence	
A-LOAM	Advanced implementation of LOAM	
AR	Augmented reality	
BoW	Bag of words	
CamVox	Camera-Livox	
CNN	Convolutional neural network	
ColorICP	Color iterative closest point	
CPU	Central processing unit	
CT-ICP	Continues-time iterative closest point	
D-LIOM	Direct LiDAR-inertial odometry and mapping	
DLT	Direct linear transform	
DOF	Degree of freedom	
DRL	Deep reinforcement learning	
DSO	Direct sparse odometry	
DTAM	Dense tracking and mapping	
DV-LOAM	Direct visual LiDAR odometry and mapping	
DVL-SLAM	Direct visual-LiDAR SLAM	
EKF	Extended Kalman filter	
EGD	Enclosed, outdoor, light-pore, feature-poor, and GPS-denied	
ESIKF	Error-state iterated Kalman filter	
ESKF	Error-state Kalman filter	
FAST	Features from accelerated segment test	
Fast-LIO	Fast LiDAR-inertial odometry	
FAST-LIVO	Fast and tightly coupled sparse-direct LiDAR-inertial-visual odometry	
FGA	Flat ground assumption	
F-LOAM	Fast LOAM	
FoV	Field-of-view	
fps	Frames per second	
g2o	General graph optimization	
G-ICP	Generalized-ICP	
GFPEAE	Geometric feature points extraction and encoding	
GICP	Generalized iterative closest point	
GPU	Graphics processing unit	
GTSAM	Georgia tech smoothing and mapping	
HDL	High-definition LiDAR	
iBoW-LCD	Incremental bag-of-words loop closure detection	
ICP	Iterative closest point	
iK-D	Incremental K-D tree	
IMLS	Implicit moving least squares	
IMU	Inertial measurement units	
iSAM2	Incremental smoothing and mapping 2	
ISC	Intensity scan context	
K-D	K-dimensional	
KF	Kalman filter	
KIO	Kinematic-inertial-odometry	
KISS-ICP	Keep-it-small-and-simple iterative closest point	
KLT	Kanade–Lucas–Tomasi	
LeGO-LOAM	Lightweight and ground-optimized LiDAR odometry and mapping	
LiDAR	Light detection and ranging	
LiLi-OM	Livox LiDAR-inertial odometry and mapping	
LIMO	LiDAR-monocular visual odometry	
LINS	LiDAR-inertial state estimator	
LIOM	LiDAR inertial odometry and mapping	
LIO-SAM	LiDAR inertial odometry via smoothing and mapping	
L-M	Levenberg-Marquardt	
LOAM	LiDAR odometry and mapping	
LOCUS	LiDAR odometry for consistent operation in uncertain settings	
LOL	LiDAR-only odometry and localization	
LSD-SLAM	Large-scale direct SLAM	
LVI-SAM	LiDAR-visual-inertial smoothing and mapping	
MAP	Maximum a posteriori	
MDC	Motion distortion correction	
ML	Machine learning	
M-LOAM	Multi-LiDAR odometry and mapping	
MSCKF	Multi-state constraint Kalman filter	
MSE	Mean square error	
MULLS	Multi-metric linear least square	
MULLS ICP	Multi-metric linear least square ICP	
NCC	Neighborhood category context	
NDT	Normal distribution transformation	
NeRF	Neural radiance fields	
OKVIS	Open keyframe-based visual-inertial SLAM	
ORB	Oriented fast and rotated brief	
OS	Operating system	
OV2SLAM	Online and versatile visual SLAM	
P2P-ICP	Point-to-plane iterative closest point	
PCA	Principal component analysis	
PGLS-LCD	Parallel global and local search loop closure detection	
PGO	Pose graph optimization	
PIN-SLAM	Point-based implicit neural-SLAM	
PnP	Perspective-n-point	
PTAM	Parallel tracking and mapping	
R2LIVE	Real-time, LiDAR-inertial-visual tightly coupled state estimator	
R3LIVE	Robust, real-time, RGB-colored, LiDAR-inertial-visual tightly coupled state estimation	
RAM	Random-access memory	
RANSAC	Random sample consensus	
RGB-D	Red-green-blue-depth	
RMSE	Root-mean-square error	
ROS	Robot operating system	
ROVIO	Robust visual-inertial odometry	
SC	Scan context	
SegMap	Segment-based mapping	
SfM	Structure from motion	
SIFT	Scale-invariant feature transform	
SLAM	Simultaneous localization and mapping	
S-MSCKF	Stereo multi-state constraint Kalman filter	
SOFT-SLAM	Stereo odometry feature tracking SLAM	
S-PTAM	Stereo parallel tracking, and mapping	
STCM	Space-time circle matching	
SuMA	Surfel-based mapping	
SuMA	Surfel-based mapping	
SURF	Speeded-up robust features	
SVO	Semi-direct visual odometry	
TSDF	Truncated signed distance function	
UKF	Unscented Kalman filter	
V-GICP	Voxelized generalized iterative closest point	
VINS	Visual-inertial system	
VIO	Visual-inertial-odometry	
VIORB	Visual-inertial ORB SLAM	
VR	Virtual reality	
WIO	Wheel-inertial-odometry	
X-ICP	Extreme-ICP	
YOLO	You only look once	
**Method**	**Symbols**	**Meaning**
LOAM	Subscription k	Counter of weeps
	P_k_	Point cloud
	L	LiDAR coordinate system (xyz)
	W	World coordinate system (xyz)
	$X_{k,i}^{L}$	Coordinates of point i in L
	$X_{k,i}^{W}$	Coordinates of point i in W
	i,j	Counter of points
	S	Set of consecutive points in a scan
	c	Local surface smoothness
	$\bar{P_{k}}$	Projected point cloud into sweep k-end time
	$\bar{Q_{k}}$	Projected point cloud into W
	t	Timestamp
	ε	Edge points
	H	Planar points
	d_ξ_	Point-to-line distance
	d_h_	Point-to-plane distance
	T	Pose transformation matrix
	λ	Constant
	f	Set of feature points
	d	Distance
IMLS-SALM	P_k_	Point cloud
	k	Counter of point clouds
	P_i_	Point i
	i	Counter of points
	n	Normal of a point
	H	Surface definition parameter
	$W_{t}^{w}$	True pose
	$W_{u}^{w}$	Update transformation
	$W_{e}^{w}$	Odometry pose estimation of the robot
LOCUS	$\hat{T}$	Scan-to-scan optimal transformation
	$\overset{-}{T}$	Scan-to-submap optimal transformation
	ϵ	Residual error
	L_k_	LiDAR scan
	K	Time step
	T	Transformation matrix
	S_k_	Local submap
Multiverse Odometry	M	Source point cloud
	N	Target point cloud
	M	Point in source point cloud
	D	Minimum distance between m and its nearest neighbor point in N
	D_chamfer_	Chamfer distance
EKF	x	State vector of the system
	v	Noise
	A	A matrix that the number of columns and rows are equal to the number of columns and rows for the state vector
	t	Timestamp
	B	A matrix that represents how the state of the system changes from t−1 to t as a result of control’s commands
	y	Predicted various sensor measurements
	H	A matrix to convert the state at time t into predicted sensor observations at time t
	w	Error matrix of the sensor
	$P_{t|t-1}$	Covariance matrix
	Q_t_	Action uncertainty matrix
	z	Observation vector
	R	Measurement noise covariance matrix
	S	Measurement residual covariance
	$\tilde{y}$	Residual measurement
	K	Kalman gain
	$\hat{x}$	Corrected state estimate
	$\hat{y}$	The updated value of the y
